# Systematic Investigation on Particle and Pore Size Effects of Mesoporous Silica on Molecular Mobility and Physical Stability of Overloaded Amorphous Acetaminophen

**DOI:** 10.1021/acs.molpharmaceut.6c00402

**Published:** 2026-08-24

**Authors:** Francisca Larasati, Kohsaku Kawakami

**Affiliations:** † Research Center for Macromolecules and Biomaterials, 52747 National Institute for Materials Science, 1-1 Namiki, Tsukuba, Ibaraki 305-0044, Japan; ‡ Graduate School of Science and Technology, University of Tsukuba, 1-1-1 Tennodai, Tsukuba, Ibaraki 305-8577, Japan

**Keywords:** Amorphous, Crystallization, Cooperatively Rearranging Region, Mesoporous Silica

## Abstract

Polymeric excipients are generally used for physical stabilization of amorphous drugs. However, a more strategic design of amorphous formulations is needed to reduce the amount of excipients, for which the use of mesoporous materials can be a promising option. In this study, mesoporous silica (MS) with various particle and pore sizes were added to amorphous acetaminophen (AAP) at various mixing ratios to systematically clarify their stabilization mechanisms. In particular, how the presence of MS affects the stability, at mixing ratios where AAP is present in excess relative to the pore volume, was focused. Six types of MS, with pore and particle sizes ranging from 2.5 to 21 nm and from 3 to 9 μm, respectively, were used in the study. Differential scanning calorimetry (DSC) analysis suggested the presence of three molecular fractions of amorphous AAP with different molecular mobilities: free, intermediate, and rigid fractions. The free fraction behaved similarly to pure AAP, whereas the intermediate fraction appeared to be relatively more stable. The rigid fraction had limited molecular mobility and was expected to strongly interact with the MS surface. AAP molecules mainly existed as the free fraction in the presence of 10% MS, regardless of the MS type. The free fraction disappeared to allow domination by the intermediate fraction with 50% MS having 17 or 21 nm pores, whereas a significant amount of the free fraction remained with MS having 2.5 nm pores. Broadband dielectric spectroscopy (BDS) analysis revealed that the addition of MS both increased and decreased the molecular mobility of amorphous AAP. The presence of all types of 10% MS slightly accelerated the γ-relaxation of amorphous AAP without affecting the α-relaxation. The addition of 50% MS with 17 or 21 nm pores slowed down the *a*-relaxation time of amorphous AAP, whereas the effect was marginal for MS with 2.5 nm pores. Isothermal crystallization of amorphous AAP at 60 °C was accelerated in the presence of MS with 2.5 nm pores at mixing ratios of both 10 and 50%. In contrast, 50% MS with 17 or 21 nm pores exhibited a strong stabilization effect. The accelerated crystallization in the presence of MS with 2.5 nm pores was explained by an increase in local molecular mobility. Mixtures with MS with 17 or 21 nm pores exhibited higher stability, which was explained by the large portion of the intermediate fraction with slower motion relative to that of pure AAP. The intermediate fraction was assumed to move inside and outside the pores, and the ease of molecular exchange was explained by the size of the pores and characteristic length of the cooperatively rearranging region of amorphous AAP. These observations provide guidance for the selection of MS materials for the stabilization of large amounts of pharmaceutical glasses and the scientific significance of the molecular dynamics of glasses in the presence of porous materials.

## Graphical abstract

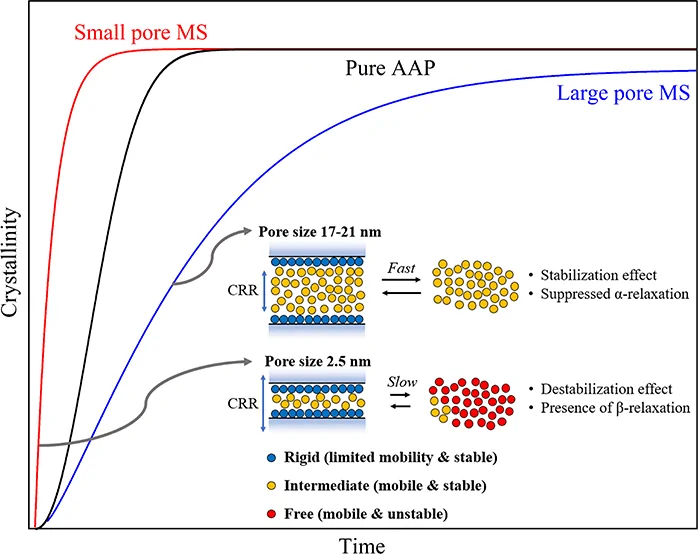

## 1 Introduction

Enhancing the oral absorption of drugs with poor water solubility is a major pharmaceutical challenge because low solubility often results in poor bioavailability.^1^ One effective approach to overcome this issue is to convert a drug from a crystalline to an amorphous state.^1–3^ The amorphous state has a higher internal energy owing to the absence of long-range ordering and thus has higher solubility relative to its crystalline counterpart;^4,5^ therefore, this may improve oral absorption in the human body.^5^ However, improving the physical stability of the drugs is essential for preventing recrystallization upon contact with aqueous media and during storage.^6–9^ Numerous studies have identified various factors that influence the recrystallization of amorphous drugs, with molecular mobility often highlighted as a key contributor.^10–13^ Therefore, reducing the molecular mobility can effectively slow down or prevent crystallization.

One method to address this challenge is the addition of mesoporous materials,^14–16^ in which two mechanisms are generally assumed to contribute to the stabilization. The first mechanism is the confinement effect. Crystal nuclei cannot form if the pore size is smaller than the critical nuclei size. Moreover, the limited space is expected to restrict molecular mobility, which plays a crucial role in retarding crystallization.^17^ The second mechanism involves molecular-level interactions (e.g., hydrogen bonding) between the drug and material surface.^18–20^ The stabilization effect depends significantly on the pore size, regardless of whether the amount of drug is greater or less than the loading limit of the pores. Most research on this subject has been conducted below the loading limit. For example, a pore-size-dependent stabilization effect was observed for amorphous menthol loaded into MSs with pore sizes of 3.2 and 5.9 nm, as demonstrated by the increased glass transition temperature (*T*_g_).^21^ A dielectric spectroscopy study revealed two relaxation processes: a faster α-process linked to the motion of bulk-like menthol molecules and a slower S-process related to menthol molecules adsorbed on the inner pore walls. Only the 3.2 nm pores prevented menthol crystallization, presumably because the pore size was smaller than the critical nucleus size.^21^ Amorphous nifedipine confined in controlled pore glass (CPG) with various pore sizes exhibited strong pore-size-dependent crystallization behavior. Crystallization was not observed for pore sizes of 7.5 and 12 nm upon heating. In contrast, nifedipine confined in larger pores (50 – 198 nm) crystallized, and a new crystal form was identified.^22^ Nuclear magnetic resonance (NMR) spectroscopy revealed that amorphous flufenamic acid remained stable for over one year in MCM-41 (3.2 nm) and SBA-15 (7.1 nm). On the other hand, flufenamic acid was crystallized in the 29 nm pores of mesostructured cellular foam (MCF), with crystallization occurring only at high drug loadings (>20 wt %).^23^ Similarly, the confinement effect of ibuprofen in MCM-41 (3.6 nm) and SBA-15 (8.6 nm) were investigated using dielectric spectroscopy. In 3.6 nm pores, crystallization was suppressed, and the α-, β_JG_-, and γ-relaxation processes deviated from bulk behavior, with a lowered *T*_g_ and τ_α_ indicating enhanced mobility. Two additional relaxation processes were identified: the S process associated with hydrogen-bonded ibuprofen at the pore wall and the D process related to interfacial dynamics. In 8.6 nm pores, the molecular dynamics were intermediate between those of the bulk solution and smaller pores.^24^

A much larger amount of excipient is required to load the amorphous drug completely into the pores, which may cause an issue of pill burden. Therefore, the drug loading larger than the pore capacity, that is overloading, is favorable from a practical point of view. In a study in which ibuprofen was spray-dried with MS (MCM-41 and SBA-15) of various pore and particle sizes under overloaded conditions,^25^ ibuprofen was vitrified in pores smaller than 10 nm owing to spatial confinement, whereas nanocrystals formed in pores larger than 20 nm. The particle size of MS, ranging from 150 nm to 20 μm, had only a minor effect in this study. When celecoxib glass was overloaded into MS, comparison of two MSs with different pore sizes,^26^ 21 and 2.5 nm, revealed that the MS with the larger pores significantly delayed crystallization of the overloaded celecoxib glass, while faster crystallization occurred with the MS with the smaller pores. These studies revealed that the stabilization effect of pores is effective for molecules outside as well as those inside the pores. The ease of exchange of drug molecules inside and outside the pores likely explains the different stabilization effects depending on the pore size of MS. In the celecoxib study, the MS pore size that could effectively stabilize the glassy state was larger than the characteristic length of the cooperative rearranging region (CRR) of the celecoxib glass but that failed to stabilize the glassy state was smaller. In the study where MSs with different particle sizes (2.5–3.7 and 59 μm) were used to stabilize overloaded simvastatin glass,^27^ only the smaller particles exhibited a strong inhibitory effect on recrystallization. This may also be explained by the difference in the molecular exchange inside and outside the molecules, as the surface area available for exchange was significantly different for both particles.

From a formulation perspective, the use of porous materials has been demonstrated to improve the storage stability and release behavior of amorphous drugs. For example, celecoxib glass remained in amorphous state for at least 18 months when formulated with MS and stored at 40 °C under a dry condition if the drug was loaded below the loading limit, which was much better stability than that of pure amorphous celecoxib. However, recrystallization was observed at 75% RH, regardless of drug loading, which was explained by the replacement of celecoxib molecules on the MS surface with water, leading to recrystallization outside the MS pores.^28^ Itraconazole confined in SBA-15 remained amorphous for one year at 4 and 25 °C. Meanwhile, the supersaturation behavior during dissolution in simulated gastric fluid was affected by the storage humidity. Storing the formulation at 52% and 97% RH beforehand improved the release rate of the loaded molecules. This effect was linked to the increased hydroxylation of the SBA-15 surface under humid conditions, which enhanced hydrophilicity and facilitated better wetting, leading to improved itraconazole release.^29^ Overloaded amorphous indomethacin with MCM-14 and SBAFr-15 was physically stable for 3 months under stressed conditions (30 °C/56% RH), where thermal analysis detected less than 3 wt % crystalline indomethacin. Although use of MCM-14 exhibited chemical stability issues for amorphous indomethacin, this was not the case for SBA-15.^30^ These observations indicate the usefulness of porous materials as excipients of amorphous formulations. However, systematic understanding on the stabilization effect has not been available.

This study provides a systematic investigation of the stabilization of amorphous acetaminophen (AAP) by using MSs with various particle and pore sizes. Although AAP does not have serious low-solubility or oral absorption issues, detailed information on its crystallization behavior^31^ is helpful for understanding the stabilization effect of porous materials in a systematic manner. The molecular mobility and crystallization behavior of amorphous AAP were analyzed by using differential scanning calorimetry (DSC) and broadband dielectric spectroscopy (BDS) to determine the influence of the particle and pore sizes of MS on the molecular dynamics and physical stability of an amorphous drug.

## 2 Materials and Methods

### 2.1 Materials

AAP with a molecular weight (*M*_W_) of 151.16 g/mol was purchased from MP Biomedicals (Solon, Ohio, USA) and used without purification. Six types of MS with various particle and pore sizes were obtained from Fuji Silysia Chemical (Kasugai, Japan). The specifications of MSs are listed in Table 1.

**Table 1:** MS Specification

Trade Name	Particle Size (μm)	Pore Size (nm)	Surface Area (m^2^/g)	Pore Volume (mL/g)	Abbreviation
Sylysia 320	3.2	21	300	1.6	D3P21
Sylysia 370	6.4	21	300	1.6	D6P21
Sylysia 380	9	21	300	1.6	D9P21
Sylysia 440	6.2	17	350	1.25	D6P17
Sylysia 730	4	2.5	700	0.44	D4P2
Sylysia 770	6.7	2.5	700	0.44	D6P2

### 2.2 Preparation of MS/AAP Physical Mixtures

Binary mixtures of AAP and MS were prepared by physically mixing AAP and MS in various ratios for 20 min by using a mortar and pestle. The mixing ratios are expressed as the percentage of MS. Silica materials are abbreviated by their diameter and pore size, as shown in Table 1. For example, a mixture of AAP and Sylysia 320 at a weight ratio of 9:1 is expressed as AAP + 10% D3P21. The grinding process had almost no effect on the particle size of the MS, as observed by scanning electron microscopy (reported in the SI).

### 2.3 Fractionation of AAP Molecules based on Molecular Mobility using DSC

DSC measurements were performed on a Q2000 (TA Instruments, New Castle, DE, USA) calibrated with indium and sapphire. Dry nitrogen was supplied as an inert gas at a flow rate of 50 mL/min. Approximately 3 mg of pure AAP or its mixtures with MS were sealed in aluminum pans. Subsequently, the samples were heated to 180 °C at a rate of 10 °C/min for melting. The temperature was maintained for 5 min, followed by cooling to 0 °C at a programmed rate of 50 °C/min. The actual cooling rate was nonlinear and slower as reported previously.^32^ To observe the glass transition, cold crystallization, and melting, the samples were heated again to 180 °C at a rate of 10 °C/min. Three samples of each composition were evaluated.

The fractionation of amorphous AAP molecules into rigid, intermediate, and free fractions was performed according to our previously published protocol.^26^ Briefly, molecules that did not exhibit a glass transition were classified as the rigid fraction, which was determined by the reduction of Δ*C*_p_ relative to pure amorphous AAP. The molecules that crystallized during DSC heating were called the free fraction, which was calculated from the cold crystallization enthalpy. The remaining molecules were attributed to the intermediate fraction, which exhibited a glass transition but could not crystallize during DSC heating; therefore, this fraction had higher stability against crystallization than the free fraction. It should be stressed that this fractionation is basically a phenomenological classification based on the DSC observation. Physical state of each fraction may depend on experimental systems.

### 2.4 Isothermal Crystallization Study on DSC

The isothermal crystallization study was conducted at 60 °C in DSC. The crystallinity was calculated under assumption that Δ*C*_p_ is proportional to the remaining mobile parts of the amorphous fraction, i.e., the sum of the intermediate and free amorphous fractions. Approximately 3 mg of AAP and its mixtures with MS were loaded and sealed in aluminum pans and heated to 180 °C at a rate of 10 °C/min for melting. The samples were cooled to −10 °C at 50 °C/min, followed by heating to 60 °C at 10 °C/min. Subsequently, the samples were annealed at this temperature for predetermined periods. Then, the samples were cooled to – 10 °C at 50 °C/min and heated again to 180 °C at 10 °C/min to observe the glass transition behavior. Three different samples were used for the measurements.

### 2.5 Physical Characterization Using X-ray Powder Diffraction (XRPD)

After cold crystallization in the DSC study, the samples were subjected to XRPD analysis to confirm the crystal forms of AAP. The DSC heating at 10 °C/min was terminated at 110 °C to collect the samples from DSC pans. XRPD patterns were acquired on a Rigaku RINT Ultima X-ray diffraction system (Rigaku Denki, Tokyo, Japan) with Cu Kα radiation at an acceleration voltage of 40 kV and a current of 40 mA. A scan rate of 2 °C/min with 0.02° intervals was used over a 2θ range from 3° to 40°.

### 2.6 Density Measurement

The true densities of AAP, MS, and their mixtures in the amorphous state were determined using an AccuPyc II gas pycnometer (Micromeritics, Norcross, GA, USA) using helium gas. Samples were melted on a hot plate at 200 °C for 10 min and left at 25 °C for cooling in an airtight box to prevent moisture uptake. The MS does not melt; therefore, its homogeneous dispersion was visually confirmed. Subsequently, the samples were milled using a hand-shaken agate mill with liquid nitrogen and dried in a vacuum oven at 40 °C for 30 min. The MS density was measured using an as-is material. The measurements were repeated ten times to obtain the mean and standard deviation values.

### 2.7 Investigation of the Molecular Mobility Using BDS

The molecular mobility of AAP and its mixtures with MS were evaluated using an Alpha dielectric spectrometer (Novocontrol, Montabaur, Germany). The samples were loaded between two parallel plates made of stainless steel with a diameter of 20 mm, and the thickness was controlled to 0.1 mm using silica spacer fibers. The samples were melted at 200 °C for 10 min on a hot plate, followed by cooling at room temperature to acquire the glassy state. The samples were mounted to the instrument immediately and heated at 190 °C for 15 min to remove residual moisture. The temperature was controlled by a Quattro temperature controller with a temperature deviation smaller than 0.2 °C. The dielectric experiments were conducted in a frequency range from 10^–1^ to 10^6^ Hz and temperature range from −125 to 65 °C, with a step of 5 °C. Two different samples were evaluated to confirm reproducibility. The dielectric spectra were fitted using the following Havriliak–Negami (HN) function:^19,33^

1 $$\varepsilon^{*}\left(\omega \right)=\varepsilon^{\prime }\left(\omega \right)-i\varepsilon^{\prime \prime }\left(\omega \right)=\varepsilon_{\infty }+\underset{k}{\sum }\frac{\Delta \varepsilon }{{\left[1+({i\omega \tau_{\mathrm{HN}})}^{a}\right]}^{b}}$$

In the equation, ε*­(ω) is the complex dielectric permittivity; ε′(ω) and ε″(ω) are real and imaginary parts of the complex permittivity, respectively; ε_∞_ is the high-frequency limit permittivity; Δε is dielectric strength; ω is the angular frequency (ω = 2π*rf*; where *f* denotes frequency); τ_HN_ is the relaxation time; and *a* and *b* are exponents of the relaxation processes. The relaxation time was determined using the following equation:

2 $$\tau_{i}=\tau_{\mathrm{HN}}\times {\left[\sin\left(\frac{\pi ab}{2+2b}\right)\right]}^{1/a}{\left[\sin\left(\frac{\pi a}{2+2b}\right)\right]}^{-1/a}$$

where *i* may be α, β, or γ for describing the *i*-relaxation time.

### 2.8 Observation of Crystal Growth under Polarized Light Microscopy (PLM)

AAP crystal growth was investigated using a PLM (Olympus BX-51, Tokyo, Japan) equipped with a U-POT polarizer and a U-ANT analyzer. A Linkam 10083L system (Linkam, Salford, UK) was used to control the sample temperature. The accuracy of the temperature control was confirmed to be ± 1 °C by evaluating the melting temperatures of standard compounds. Pure AAP and its mixtures with 10% D3P21 and 10% D4P2 were melted on a thin glass slide at 200 °C using a hot plate. A cover glass was then placed over the samples, which were subsequently cooled by leaving them at 25 °C in an airtight box to prevent moisture uptake. The absence of crystals was confirmed before observation. Crystal growth was investigated at 70, 80, 90, and 100 °C.

### 2.9 Calculation of the Size of the Cooperatively Rearranging Region (CRR)

The size of the CRR was calculated by α-relaxation times obtained from BDS measurements. Based on Adam–Gibbs theory, molecular dynamics in glassy systems are governed by cooperative rearrangements, allowing the size of CRR to be quantified. Since nucleation is associated with cooperative molecular motion, the CRR size of amorphous AAP was calculated using the following equation:^34–36^

3 $$Nc=\frac{R}{{\Delta C}_{p}m}{\left(\frac{\beta }{e}\right)}^{2}{\left(\frac{d\ln\tau_{\alpha }}{d\ln T}\right)}^{2}$$

where *Nc* is the number of molecules with cooperative motion. τ_α_ and β are α-relaxation time and parameter describing its distribution, respectively. Δ*C*_p_, *m*, and *e* represent the heat capacity change at *T*_g_, molecular weight, and Euler’s number, respectively. *R* is gas constant.

## 3 Results

### 3.1 Fractionation of Amorphous AAP in its MS Mixtures

The first DSC heating curves for crystalline AAP and its mixtures with 25% MS are shown in Figure S1. During this stage, the melting behavior of samples mixed with MS having pore sizes of 17 and 2.5 nm was the same as that of pure AAP, whereas two melting peaks were observed for the mixtures with MS having a pore size of 21 nm. AAP crystals are expected to be partially vitrified and penetrate pores during the grinding process^37^ when the pore size is large. The appearance of a melting peak at low temperatures indicates the Gibbs–Thomson effect in the pores. Figure 1a shows the second heating curves of the samples with 25% MS, which were obtained after cooling to −10 °C. All samples exhibited *T*_g_ at 24 °C, followed by three types of cold crystallization and transformation behaviors. Figure 2 shows the XRPD patterns of the samples collected at 110 °C, for which a detailed explanation will be provided later. In the case of pure AAP, cold crystallization resulted in form III, followed by a solid-state transformation to form II and its melting.^38^ However, in the presence of MS with 21 nm pores, the transformation from form III to form II followed a melt–crystallization pattern, which was most likely due to the decrease in the melting temperature of form III based on the Gibbs–Thomson effect. The behavior with MS having a pore size of 17 nm was between the two patterns mentioned above. However, cold crystallization proceeded directly into form II in the presence of MS with 2.5 nm pores. An investigation using PLM suggested an immediate transformation from form III to form II during cold crystallization. Therefore, the presence of MS with small pores facilitated the crystallization process. An increase in the amount of MS to 50% reduced the Δ*C*_p_ for all samples (Figure 1b), suggesting an increase in the rigid fraction. The mixtures with 2.5 nm pores, D4P2 and D6P2, showed a cold crystallization peak followed by melting behavior. A small melting peak with an enthalpy smaller than 3 J/g was observed in a reproducible manner for D6P17 and D3P21, and this peak was found at a slightly lower temperature (145 °C) than that of pure AAP (157 °C). These peaks are also understood as a result of cold crystallization, although exothermic peaks originated from cold crystallization are not visible because of their smallness.

**Figure 1:**
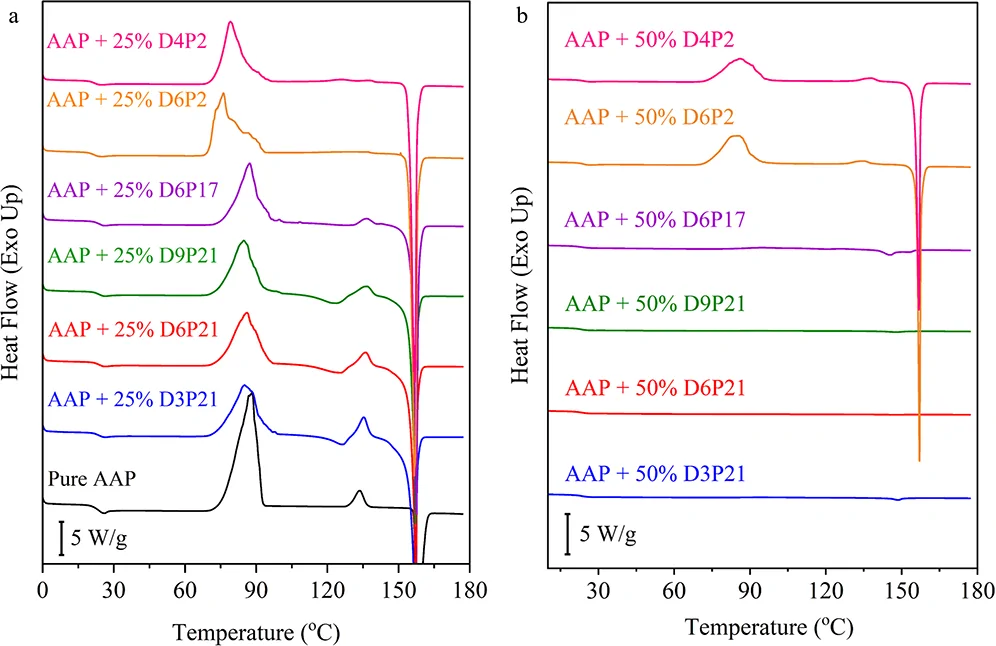
(a) The second heating DSC curves for pure AAP, its mixture with 25% MS, and (b) its mixture with 50% MS.

**Figure 2:**
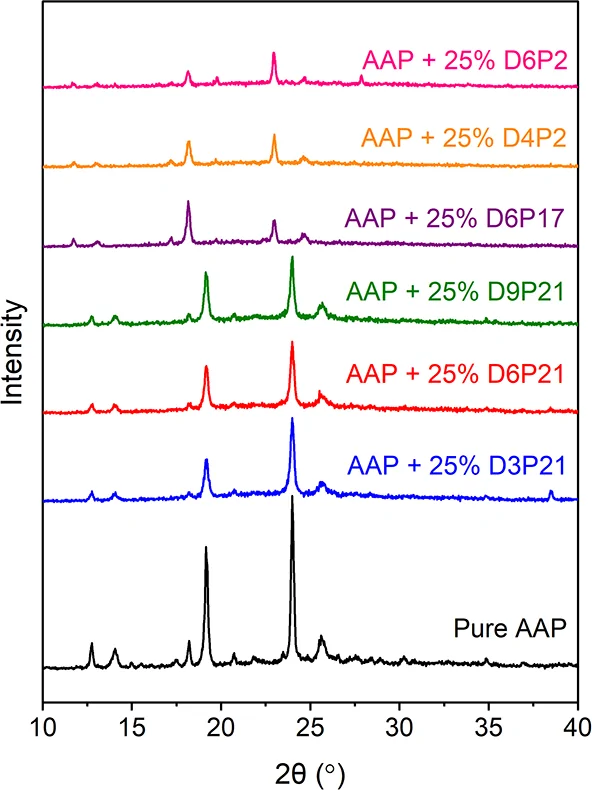
XRPD patterns of pure AAP and its mixtures with 25% MS after recrystallization during DSC. The samples were collected from DSC pans after heating was terminated at 110 °C.

AAP exhibits three major crystal forms: stable form I (monoclinic), metastable form II (orthorhombic), and form III. The strong diffraction peak at 24° suggested the formation of form III, which was found after the cold crystallization of pure AAP and its mixtures with MSs with 21 nm pores. In contrast, cold crystallization to form II was observed for other mixtures (Figure 2). The DSC curve (Figure 1a) indicated that a small portion of form III remained at 110 °C but likely disappeared during sample preparation for XRPD evaluation.

Figure 3a shows the proportion of each fraction of amorphous AAP at different mixing ratios with MS. For all MS types, the rigid fraction increased with increasing MS content. Meanwhile, particle size variation showed minimal influence. Up to 50% MS, the rigid fraction remained relatively low for MSs with 21 nm pores, whereas MSs with 2.5 nm pores exhibited a larger rigid fraction. The intermediate fraction dominated in the presence of 50–70% MSs with 21 nm pores. In contrast, a notable amount of the free fraction was retained in the presence of MSs with 2.5 nm pores even at 60% MS, which disappeared at 70%. In summary, 17 or 21 nm pores effectively converted the amorphous AAP molecules into the intermediate fraction even under significantly overloaded conditions, whereas this effect was weak for MSs with 2.5 nm pores.

**Figure 3:**
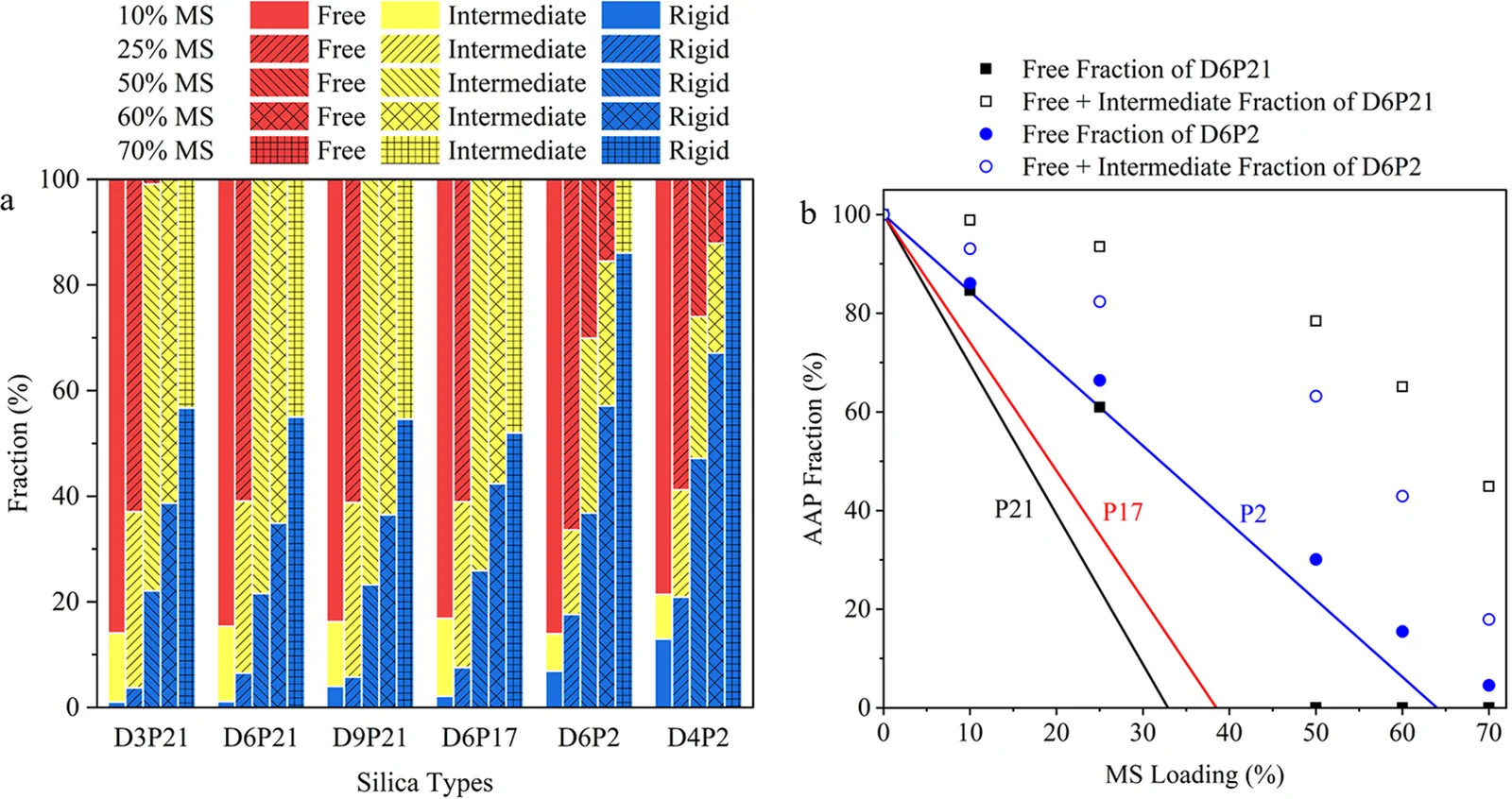
(a) Fractionation of amorphous AAP in the Mixtures with MSs. (b) Fraction of AAP remaining outside the pore as a function of MS loading. Solid lines show theoretical calculations using the pore volume of each MS and the density of AAP. P21, P17, and P2 correspond to MSs with pore sizes of 21, 17, and 2.5 nm, respectively. The *x*-axis intercepts indicate the MS loading required to completely accommodate all AAP within the pore. The free fraction and the sum of free and intermediate fractions for D6P21 and D6P2 are also presented.

The amount of MS required to fully accommodate the AAP within the pores can be estimated by model calculations. As an example, for the P21 MSs, the MS content required to completely load the AAP molecules in the pores was calculated to be 32.9% based on the pore volume and true density of AAP (1.28 g/cm^3^). Following the same procedure, the MS contents for the complete loading were calculated as 38.5% and 64% for the P17 and P2, respectively. Figure 3b shows the theoretical AAP fraction remaining outside pores as a function of MS loading. However, an important assumption in this calculation is complete filling of the pores, i.e., it is not assumed to leave a vacancy in the pores. This was confirmed by measuring the density of each pure material and 50%AAP/MS mixtures (data not shown) to find that large pore MSs were completely filled. However, pores were found to be 34.6% and 18.4% unfilled for 50%D6P2 and 50%D4P2, respectively, indicating difficulty in filling nanosized pores. Figure 3b also shows the free fraction and the sum of free and intermediate fractions for D6P21 and D6P2. For D6P2, the theoretical fraction of AAP outside the pores was close to that of the free fraction. The slight disagreement at high MS contents can be explained by the incomplete filling mentioned above. Thus, only the molecules outside the pores are likely to behave as a free fraction. In contrast, the free fraction of D6P21 was much larger than the theoretical fraction outside the pores, despite the complete filling for this MS, indicating that some AAP molecules inside the 21 nm pores also behaved as the free fraction.

Figure 4 shows Δ*C*_p_ values as a function of the MS loading amount, which decreased linearly with its increase, reflecting an increase in the rigid fraction. Linear extrapolation to Δ*C*_p_ = 0 provides the amount of MS required to transform all AAP molecules into the rigid fraction (Table 2). Although the suppression effect of cold crystallization was weak for MSs with 2.5 nm pores (Figure 1b), the proportion of the rigid fraction was larger for these MSs. This can be explained by the large surface area of MSs with 2.5 nm pores under the assumption that monolayered molecules on the surface behave as a rigid fraction.

**Figure 4:**
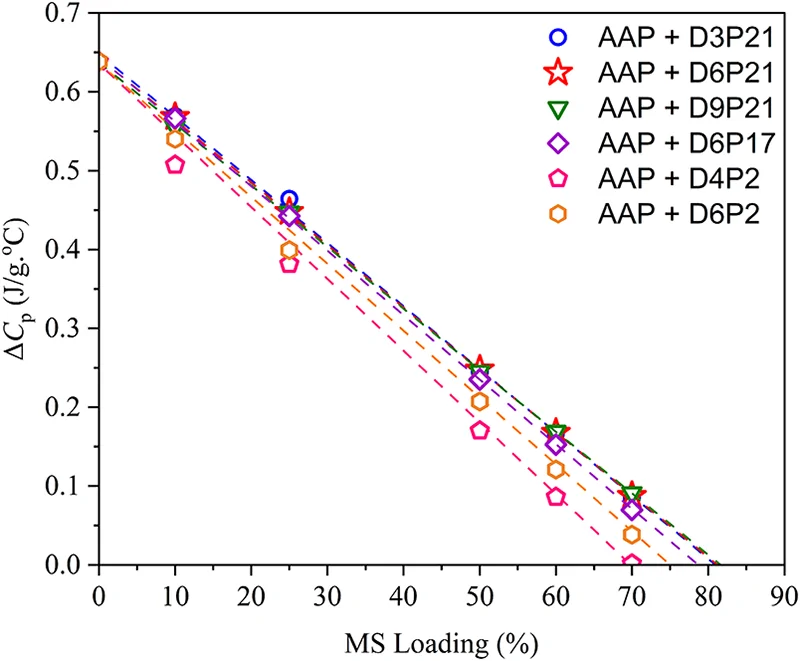
Change in the Δ*C*_p_ of AAP as a function of MS loading.

**Table 2:** MS Loading Required to Erase Δ*C*_p_ of Amorphous AAP

MS	MS Loading (%)
D3P21	80.4
D6P21	80.9
D9P21	81.7
D6P17	78.4
D4P2	70.2
D6P2	74.6

The surface area occupied by each AAP molecule was calculated under the simple assumption of a cubic molecular shape and uniform density, resulting in an adsorbed area of 0.338 nm^2^ per molecule. Based on this value, the theoretical loading capacity of this MS was calculated to be 8.89 × 10^21^ AAP molecules per gram. Consequently, the MS loading required to provide sufficient surface area for monolayer adsorption was calculated to be 82%. Full conversion of AAP into the rigid fraction was achieved at 80.4% MS (Table 2), indicating that rigid fraction represents AAP molecules interacting with MS surface directly. However, although the surface area of MSs of the small pore is more than two-folds of MS with the large pores, difference in the required MS amount to erase Δ*C*_p_ is small, which can also be explained the incomplete pore filling of the small pore MSs.

Thermogravimetric analysis of pure AAP and its mixtures with MSs is reported in SI (Figure S2), where fraction of the highly stabilized AAP molecules was calculated. The values roughly agreed or slightly smaller than those of the rigid fraction determined from the DSC measurements for the mixtures with large pore MSs. However, large differences were observed for the mixtures with small pore MSs, which suggests different impacts of entrapment on physical and chemical stabilization. However, in this comparison, attention is required for difference in the temperature range for the observations. Assessment of physical stabilization is based on evaluation at *T*_g_. However, the chemical stability is investigated at the degradation temperature higher than 200 °C, where physical stabilization may have only minor impact on the chemical stability.

### 3.2 Investigation on Molecular Mobility Using BDS

Figure 5 shows the dielectric loss spectra for pure amorphous AAP (a) above and (b) below the *T*_g_, where peaks originating from *a*- and γ-relaxation were observed, respectively. The intensity of the spectra originating from α-relaxation decreased above 55 °C, indicating crystallization,^39^ because a reduction in peak intensity reflects a decrease of reorienting dipoles.^40^ The peaks shifted to higher frequencies with increasing temperature owing to an increase in molecular mobility. These observations are consistent with literature reports.^19,21,27^ All the spectra fit well with the HN function.

**Figure 5:**
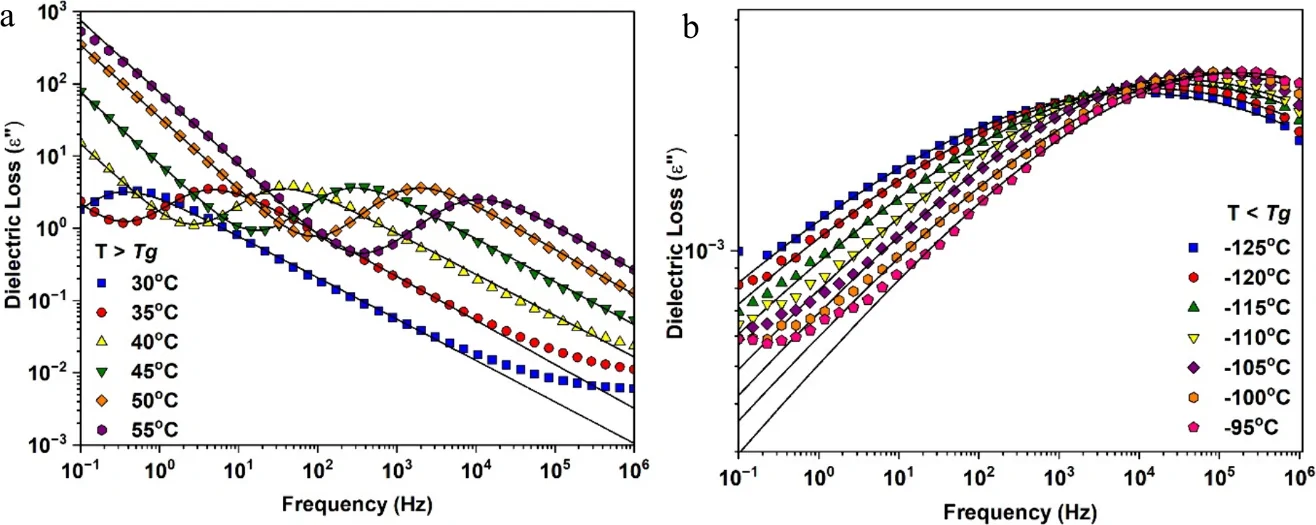
Dielectric loss spectra of pure amorphous AAP in temperature ranges (a) above and (b) below *T*_g_.

Figures 6–8 show examples of the dielectric loss spectra of amorphous AAP mixed with 10% MSs. All spectra for mixtures with 10, 25, 50, 60, 70 and 80% MSs are provided in the SI. The intensity of the spectra originated from α-relaxation decreased above 60 °C for the mixtures with 10% MSs due to crystallization (Figure 6), indicating better physical stability than pure AAP. However, the locations of the peaks originating from α-relaxation were not affected by MS addition, indicating no effect on τ_α_.

**Figure 6:**
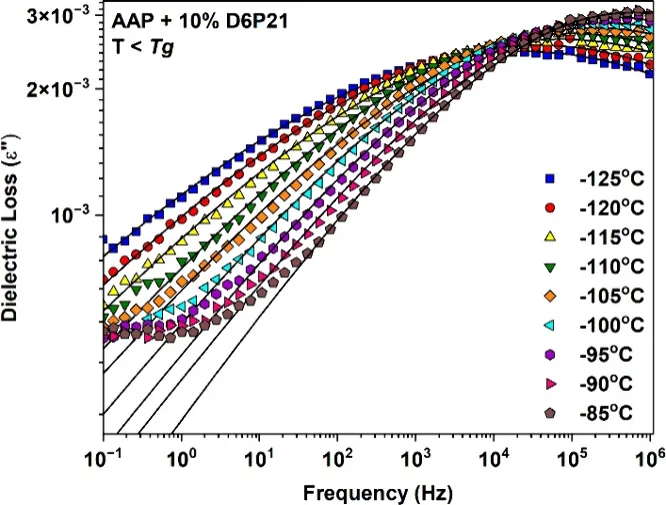
Dielectric loss spectra of amorphous AAP mixed with 10% MSs, having (a) small pores (D6P2) and (b) large pores (D6P21), measured at temperatures above *T*_g_ to investigate α-relaxation.

As for secondary relaxations, γ-relaxation was clearly visible for both pure AAP and 10% MS mixtures, which slightly moved to higher frequency upon the addition of MSs (Figure 7 and 8b). Although peaks originating from β-relaxation were not visible for pure AAP, they were detected in the presence of MSs with pores smaller than 17 nm (Figure 8a), suggesting the appearance of a new mode of molecular motion upon the addition of MS with small pores.

**Figure 7:**
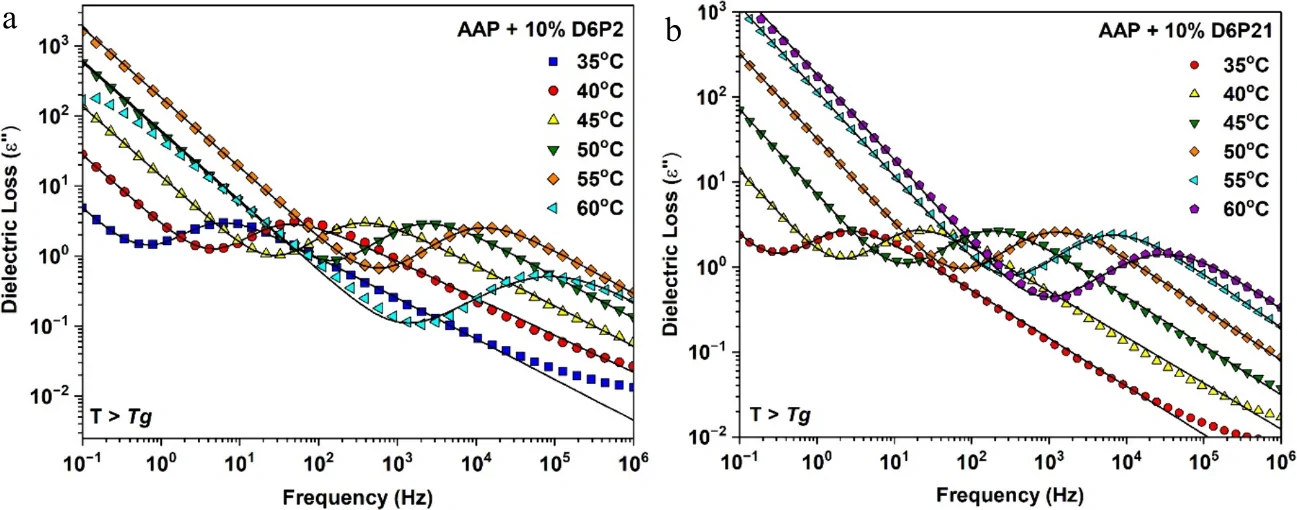
Dielectric loss spectra of amorphous AAP mixed with 10% MSs, having 21 nm pores acquired at temperatures below *T*_g_ to investigate secondary relaxation.

**Figure 8:**
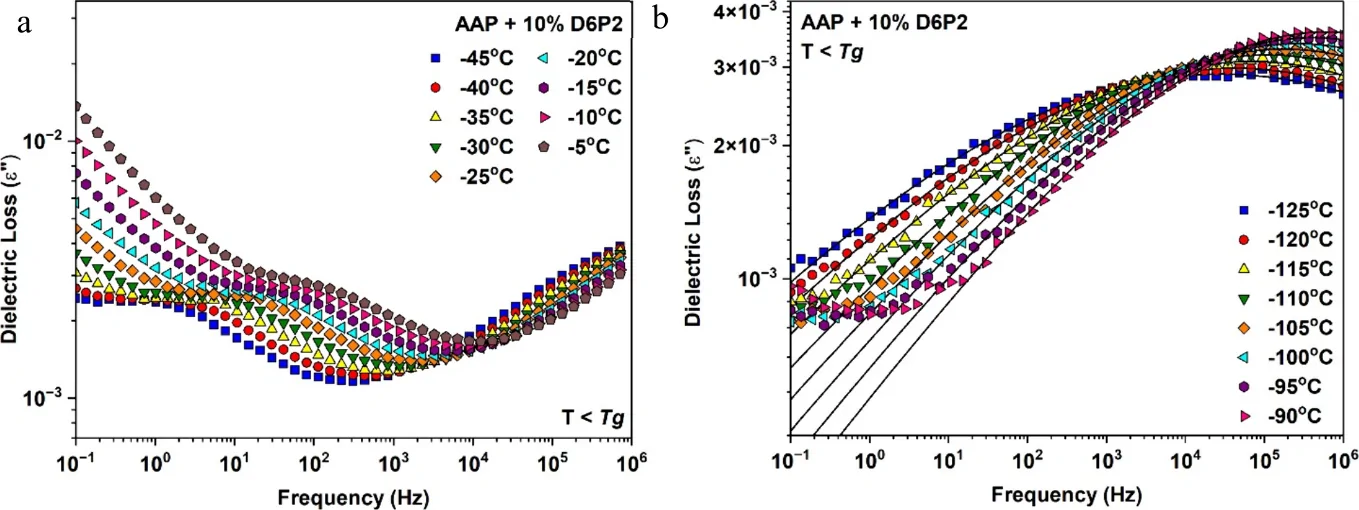
Dielectric loss spectra of amorphous AAP mixed with 10% MSs, having 2.5 nm pores (D6P2) acquired at temperatures below *T*_g_ to investigate secondary relaxation. (a) The temperature range for β-relaxation. (b) The temperature range for γ-relaxation.

Dielectric spectra were fitted using the HN function (eq 3) to determine relaxation times. In the supercooled liquid temperature region, the time-dependence of the α-relaxation time (τ_α_) was fitted using the Vogel–Fulcher–Tamman (VFT) equation:^39^

5 $$\tau_{\alpha }=\tau_{0}\exp\left(\frac{DT_{0}}{T-T_{0}}\right)$$

where τ_0_ is time scale of vibrational motion (∼10^–14^ s). *T*_0_ is the temperature analogous to the Kauzmann temperature, and *D* is the strength parameter for a measure of fragility. τ_β_ and τ_γ_ in the glassy state had a linear temperature dependence and were fitted using the Arrhenius equation as follows:

6 $$\tau_{\beta /\gamma }=A\exp\left(\frac{E_{a}}{RT}\right)$$

where *A* is a pre-exponential factor, *E*_a_ is the activation energy, and *R* is the gas constant. The temperature dependences of the α-, β-, and γ-relaxation times of AAP-MS mixtures are plotted in Figure 9. τ_α_ of amorphous AAP was well-fitted by the VFT equation and was not influenced by the addition of any type of 10% MS, with a *D* value of approximately 6. This is a natural observation because most of the AAP molecules exist as a free fraction at this mixing ratio. τ_β_ in the presence of small-pore MS followed Arrhenius behavior, with activation energies of 20.4, 46.2, and 65.5 kJ/mol for 10% D6P17, 10% D4P2, and 10% D6P2, respectively. τ_γ_ in the presence of any type of 10% MS was smaller than that of pure AAP. The activation energy for τ_γ_ of pure AAP was 23.1 kJ/mol, which was consistent with the literature value^41–43^ and not significantly influenced by the coexistence of 10% MS.

**Figure 9:**
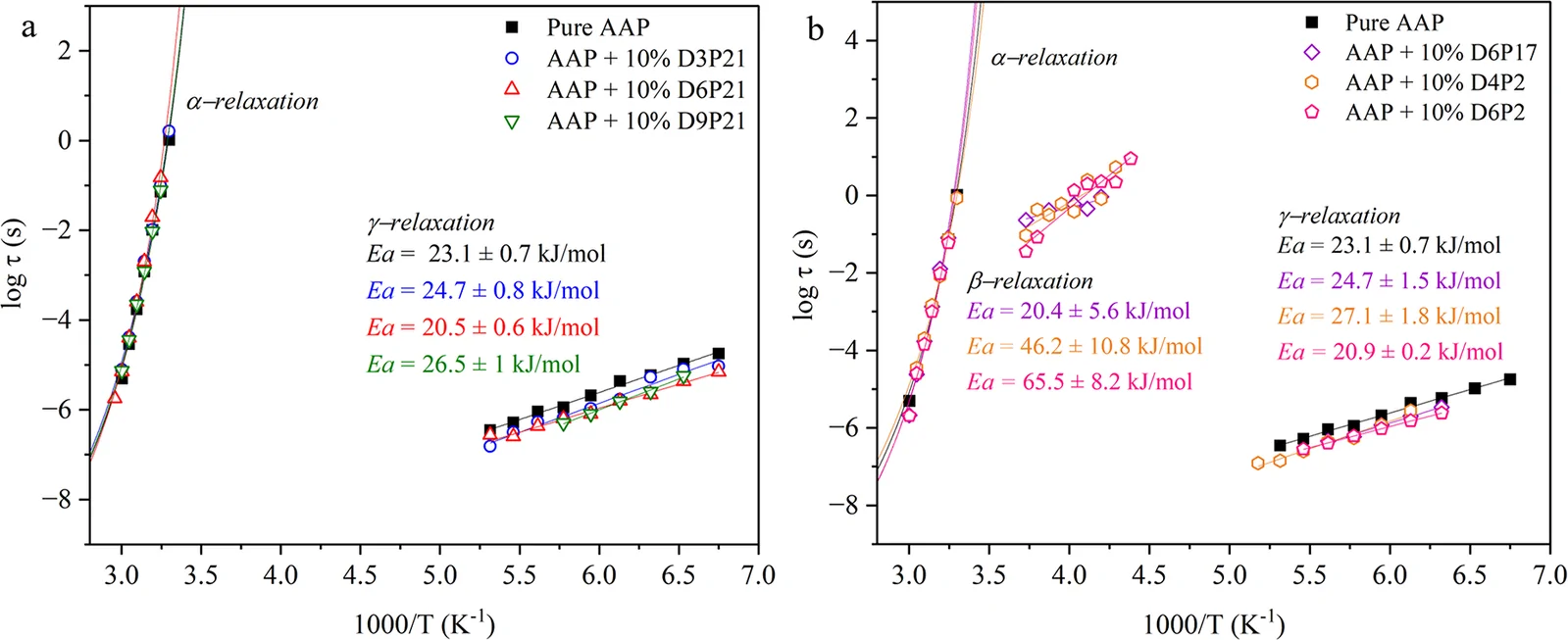
Temperature dependencies of relaxation times of pure AAP and its mixtures with MSs. (a) Pure AAP and its mixture with MSs having 21 nm pores. (b) Pure AAP and its mixtures with MSs having 17 or 2.5 nm pores. α-relaxation and secondary (β- and γ-) relaxation times were fitted with the Vogel–Tammann–Fulcher (VFT) equation and Arrhenius equation, respectively.

The impact of adding larger amount of MS on τ_α_ is shown in Figure 10. The addition of 50% MS increased the τ_α_ value of AAP. This effect was most evident with the coexistence of large-pore MS, which contained a substantial intermediate fraction. In contrast, the addition of small-pore MS rarely altered τ_α_ most likely due to the presence of the free fraction.

**Figure 10:**
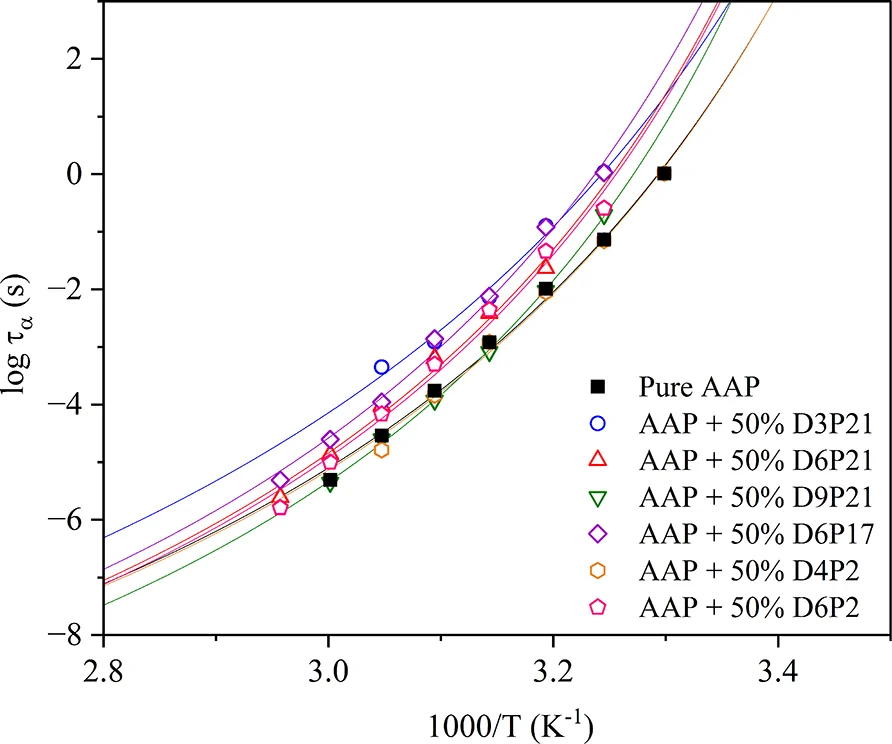
α-relaxation times of pure AAP and its mixtures with 50% MS.

### 3.3 Effect of MS on the Physical Stability of Amorphous AAP

Figure 11 shows the crystallinity evolution of amorphous AAP and its mixtures with 10% or 50% MSs at 60 °C. The data were fitted using the Avrami–Erofeev equation as follows:

7 $$X=100\left[1-\exp\left\{-k{\left(t-d\right)}^{n}\right\}\right]$$

where *k* and *d* represent the crystallization rate and induction time, respectively. *n* is the Avrami constant, which reflects the dimensions of the crystal growth and nucleation mechanism. The addition of 10% MS with 2.5 nm pores accelerated crystallization, whereas no meaningful differences were found in the presence of other types of MSs (Figure 11a). This behavior was accompanied by the appearance of β-relaxation and accelerated γ-relaxation in the small pore systems (Figure 9), suggesting a possible relationship between enhanced local molecular mobility and the accelerated crystallization. Crystallization predominantly proceeded to form II in the presence of the small-pore MSs, whereas form III was obtained for the other mixtures and pure AAP. Form I was also likely involved, as the DSC curves (data not shown) indicated melt crystallization from form II to form I. Faster crystallization was also observed in the presence of 50% small-pore MSs; however, crystallization slowed down in the presence of other types of MSs (Figure 11b). These mixtures contained large proportions of intermediate AAP fractions, which might be playing a key role in stabilizing amorphous AAP. The resultant crystal form was not influenced by the loading amounts of MSs, except that contamination of form I was not found for mixtures with 50% MSs with large pores. No tendency was detected between variation in the crystal forms and the crystallization rate, indicating that all crystal forms have the same origin, that is, nuclei.

**Figure 11:**
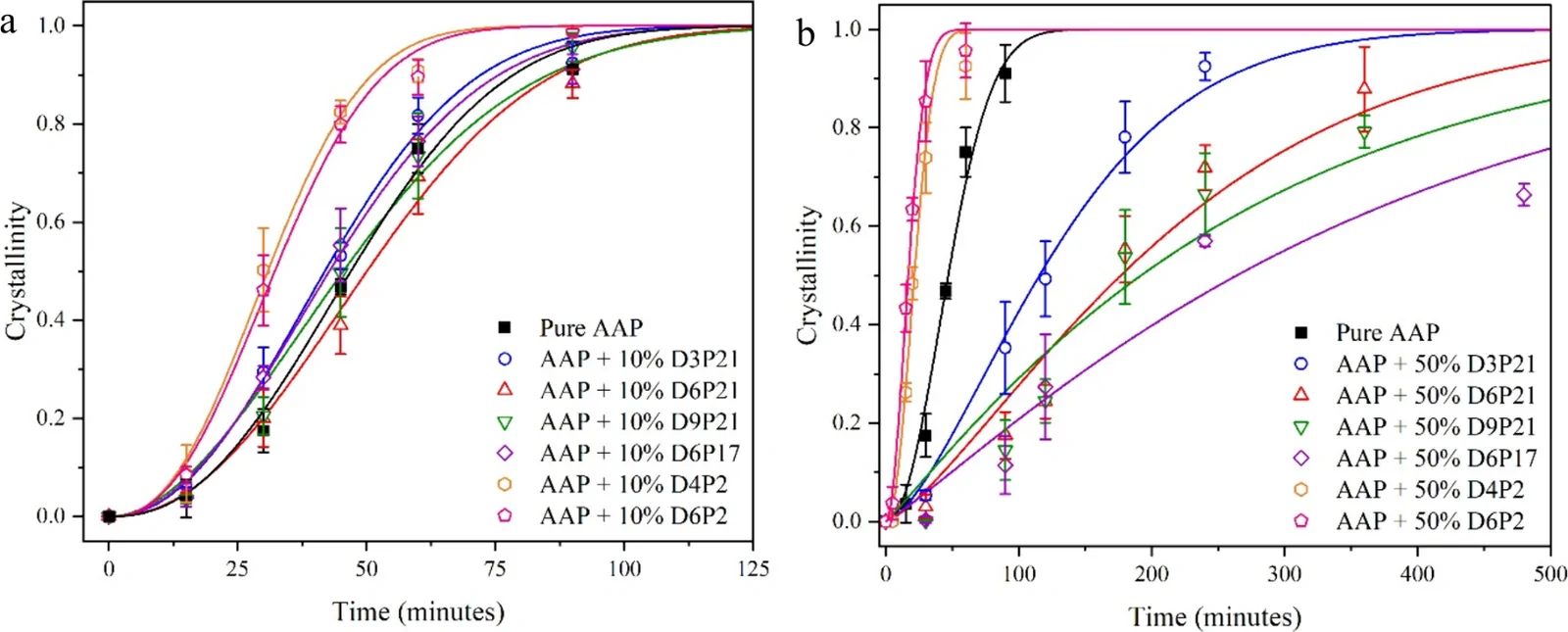
Influence of 10% MSs (a) and 50% MSs (b) on the isothermal crystallization of AAP at 60 °C. The crystallinity was calculated in reference to the sum of free and intermediate fractions.

Figure 12 shows melting enthalpy values of AAP in the DSC evaluation of crystallinity after storage for various periods at 60 °C in the presence of 50% MSs. The values did not change in the presence of MSs with small pores, indicating that only the free fraction underwent crystallization. In contrast, an increase in the melting entropy was observed in the presence of MSs with large pores, indicating that the intermediate fraction underwent crystallization.

**Figure 12:**
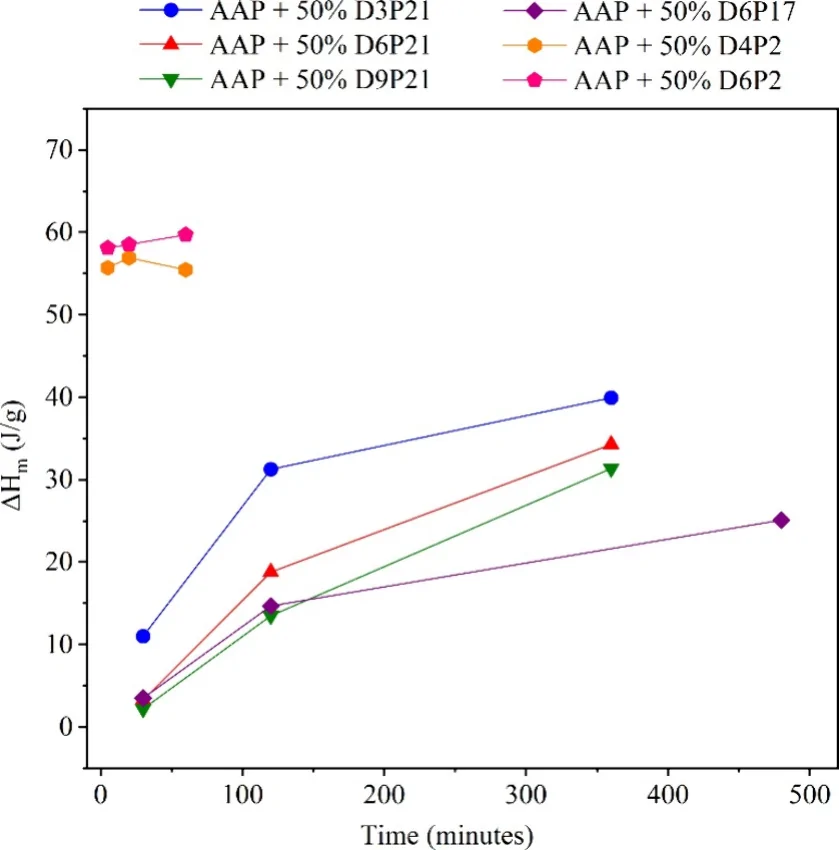
Melting enthalpy values of AAP form III in the DSC evaluation of crystallinity after storage for various periods at 60 °C with 50% MSs.

The kinetic parameters for the isothermal crystallization at 60 °C are presented in Table 3. A comparison of *k* revealed that the growth rate was slightly accelerated by adding MSs. This finding is understandable for MSs with 2.5 nm pores but does not appear to be natural for MSs with larger pores. The *n* value for the crystallization of pure AAP is ca. 2 ^17,44^ because of heterogeneous nucleation and two-dimensional crystal growth. This value was not significantly influenced by the presence of 10% MSs. However, the value decrease to 1.1–1.5 in the presence of large-pore MSs. The value for small-pore MSs did not decrease with increasing MS loading. The correlation is summarized as a figure in SI. Therefore, the intermediate fraction might be the origin of the decrease in *n*. This may be explained by a decrease in dimensions of crystal growth and/or an increase in the heterogeneity of crystallization. A similar decrease in *n* was reported for AAP confined in 103 nm pores,^45^ suggesting a change in the crystallization mechanism in the confined environment.

**Table 3:** Avrami Fitting Parameters for the Isothermal Crystallization of Pure AAP and Its Mixtures with MS at 60 °C^a^

MS	*k* (min^–1^)	*n*
None	8.56 × 10^–5^	2.3

	10% MS	50% MS	10% MS	50% MS
D3P21	1.67 × 10^–4^	5.16 × 10^–4^	2.2	1.5
D6P21	1.30 × 10^–4^	6.98 × 10^–4^	2.2	1.3
D9P21	4.70 × 10^–4^	2.42 × 10^–3^	1.9	1.1
D6P17	2.55 × 10^–4^	1.32 × 10^–3^	2.1	1.1
D4P2	1.98 × 10^–4^	8.99 × 10^–4^	2.4	2.2
D6P2	1.94 × 10^–4^	2.61 × 10^–3^	2.4	2.0

a *d* = 0 for All Samples.

### 3.4 Characteristic CRR Length of Amorphous AAP

A dynamic view is required for explaining the stabilization effect on the external molecules in the presence of porous materials. Constituent molecules in amorphous materials do not move freely but have cooperative motion. Thus, characteristic length of CRR, simply called as CRR size hereafter, is expected to have relevance with easiness of molecular exchange inside and outside pores. Two representative methods are available for determining the CRR size. The simplest method is determination from DSC data using width of the glass transition region;^46^ however, it only gives the size value at *T*_g_. Moreover, presence of molecules with different molecular mobility may broaden the glass transition region, which directly impacts the estimation. The calculated results following this procedure are presented in SI. An alternative method to obtain the CRR size is to use α-relaxation times. Figure 13 shows the CRR size as a function of temperature for each mixture calculated by α-relaxation times obtained by BDS. Despite scattering of the data, decreasing trend of CRR size with temperature was confirmed for all mixtures. For MSs with 21 nm pores, the CRR size decreased as the MS content increased, particularly at higher loadings. A similar trend was observed for D6P17. In contrast, the CRR size of 2.5 nm pores showed relatively little change with increasing MS content and remained close to that of pure AAP over most of the investigated temperature range. This difference can be explained by differences in the amount of intermediate fraction because the CRR size of the free fraction is expected to be similar to that of pure AAP. Almost all mobile molecules were assigned to the intermediate fraction when more than 50% of MSs with larger pores was added. Meanwhile, the ratios of the intermediate and free fractions were almost unchanged with an increase in 2.5 nm-pore MSs. The CRR size at 60 °C was approximately 3 nm for all mixtures, which is a little larger than the small pore but sufficiently smaller than large pores. The size determined from DSC was a little smaller but still comparable with the small pore. The CRR values obtained from BDS are generally larger compared to those calculated form DSC, because the two methods are based on different principles and probe different aspects of molecular dynamics. Relative size of pore and CRR can be one possible factor to explain the pore-size dependent stabilization effect against crystallization.

**Figure 13:**
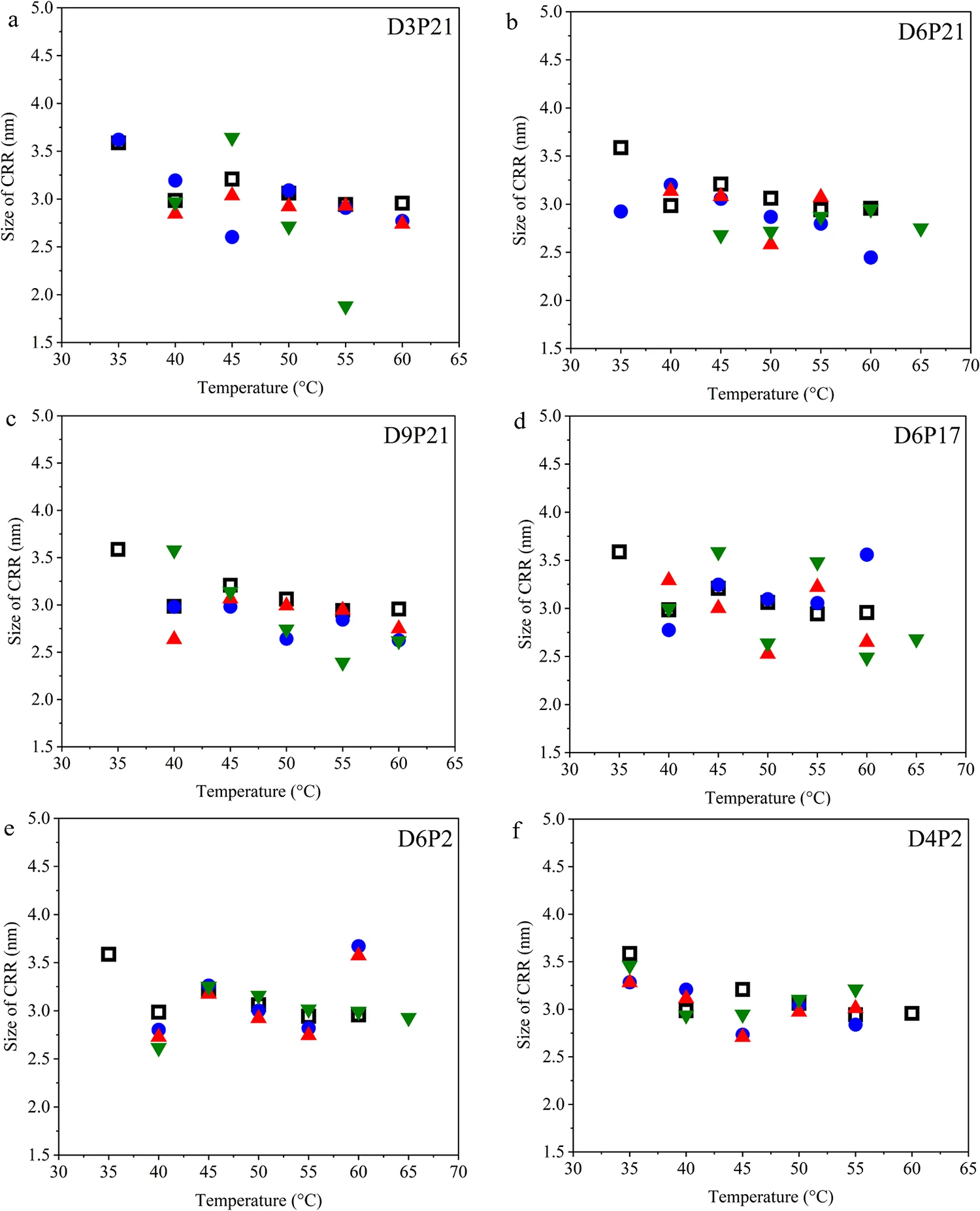
Sizes of CRR of amorphous AAP calculated from BDS. The symbols represent pure AAP (open black squares), 10% MS (blue circles), 25% MS (red triangles), and 50% MS (green inverted triangles).

### 3.5 Influence of MS on the Crystal Growth Rate of Amorphous AAP

Figure 14 a-c shows PLM images of the growing crystals at 80 °C of pure AAP and its mixtures with 10% D3P21 and 10% D4P2. Spherical crystals grew in the absence of MS and presence of D3P21, whereas the crystals formed in the presence of D4P2 exhibited a more irregular morphology, suggesting an interaction between the AAP crystals and D4P2 particles. Moreover, a larger number of crystals were always observed in the presence of D4P2, suggesting enhancement of nucleation. The crystal growth rates measured at 70, 80, 90, and 100 °C are shown in Figure 14d. The values for pure AAP exhibited reasonable agreement with reported values.^47^ Addition of 10%MSs did not provide any meaningful differences in the growth rate, which increased with increasing temperature. Thus, addition of MS is not likely to affect the crystal growth rate.

**Figure 14:**
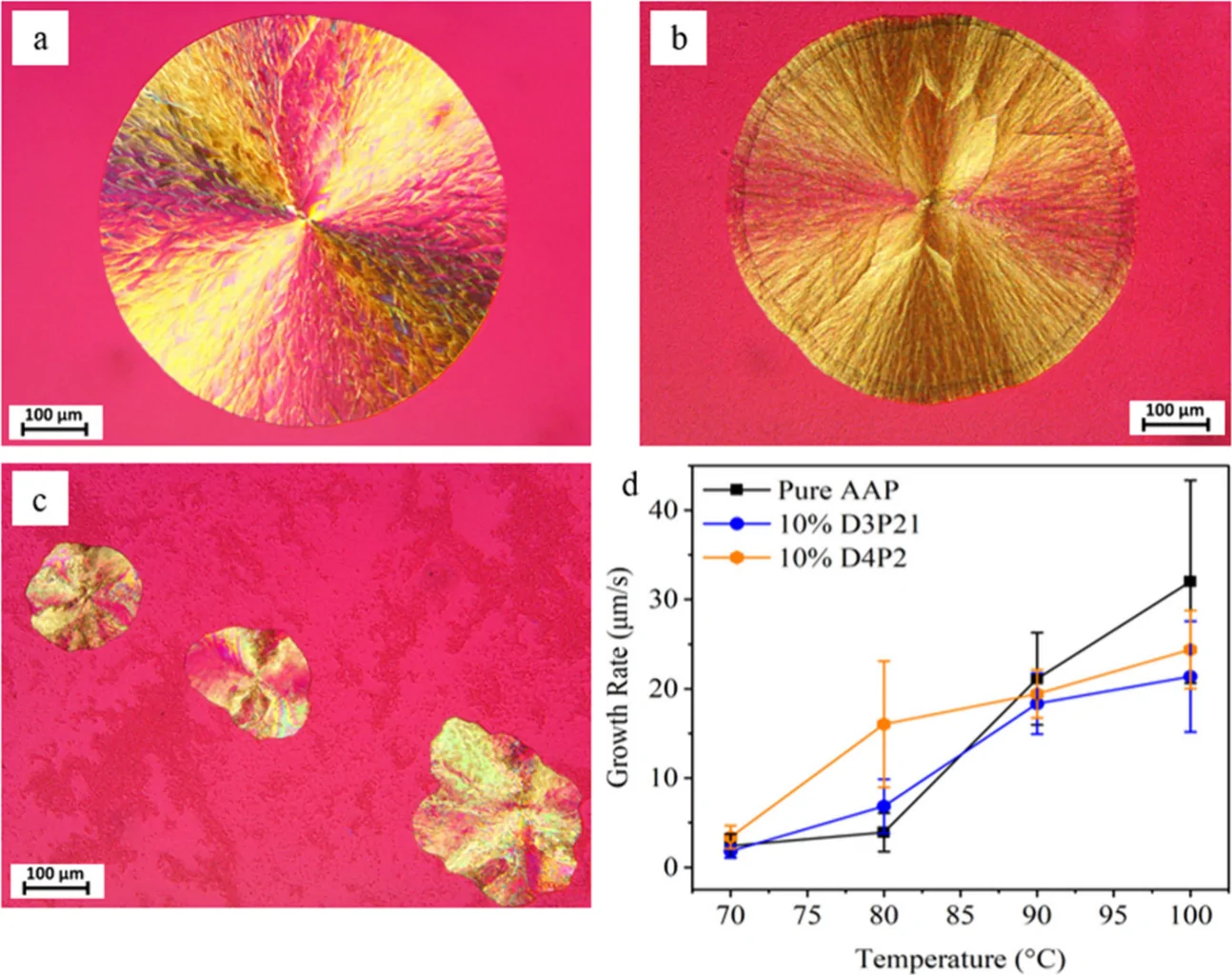
PLM images of the crystallization process of (a) AAP and its mixtures with (b) 10% D3P21 and (c) 10% D4P2 at 80 °C. (d) Crystal growth rates of AAP and its mixtures with MSs determined from PLM images at 70–100 °C.

## 4 Discussion

### 4.1 Molecular Mobility of Entrapped Molecules in Pores

The change in the molecular mobility of guest molecules in pores is one of the most important interests in the use of mesoporous carriers. Table 4 summarizes literature information on effect of entrapment of amorphous drugs into nanosized pores on their molecular mobility. Although the interaction between drug molecules and surfaces is expected to slow down the molecular mobility intuitively, increase in molecular mobility is also found frequently. The drug molecules exist in the central part of the pores are likely to be responsible for the fast dynamics. However, it also appears that minor inconsistencies still remain, which may be originated from differences in the pore sizes, interaction modes between the drug compound and pore wall, and detection methodologies. In this study, based on the BDS measurements, the α-relaxation time of amorphous AAP in the presence of MS slightly slowed down relative to that of pure AAP (Figure 10). Some earlier reports suggest no influence or decreased mobility for global motion when entrapped in pores. Amorphous aripiprazole confined in 23 nm MS pores did not influence α-relaxation, except for its distribution.^16,48^ NMR studies reported reduced mobility for indomethacin and griseofulvin in silicon material with 11.1–13.3 nm pores, while slightly increased mobility was observed at higher water content for itraconazole in SBA-15.^49,50^ However, in studies where molecules in the pores were fractionated for evaluation, molecules with higher mobility were found.^26^ The higher molecular mobility has sometimes been explained by the rearrangement of the hydrogen bonding network, which reduces the dimensions of molecular motion.^24^ The region of reduced number of the hydrogen-bonding may work as a slipping plane to increase both local and global molecular mobility. Moreover, capillary condensation may be another important mechanism for enhancing molecular mobility. The interfacial tension between the guest molecules and pore wall is a driving force that pulls the molecules parallel to the pore wall if the cross-section is completely filled. The strength of this force depends on the interfacial tension and physicochemical properties of the guest compound, including the vapor pressure, molecular mass, and density; therefore, the observation should depend on both the type of porous material and compound species. The apparent system-dependent stabilization effect may partially be explained by this factor. Among the compounds listed in Table 4, ibuprofen and AAP are examples to exhibit enhanced molecular mobility in the pores, which may be attributed to their small size and high polarity. Although menthol is also expected to offer similar observations in this scenario, the effect may be eliminated because of its high saturated vapor pressure. A wider distribution of α-relaxation times was found for various systems,^16,19,26,48^ which also indicated the presence of small portions of molecules with higher mobility.

**Table 4:** Literature Summary on the Confinement Effect on Molecular Mobility

Systems	Global Mobility	Local Mobility	Stabilization Effect	Reference
Acetaminophen/MS	Slightly decreased	Appearance of *β-*relaxation w/2.5 nm pores, faster *γ-*relaxation	Stabilized w/ >17 nm pores and destabilized w/2.5 nm pores	This Study
2.5, 17, and 21 nm pores				
Aripiprazole/MS	Unchanged α-relaxation behavior, with wider distribution at 50% MS	Unchanged *β-* and *γ-*relaxation behavior	Stabilized and destabilized depending on the mixing ratio	^48^
23 nm pores				
Aripiprazole/Al_2_O_3_, native silica, silanized silica	Unchanged α-relaxation behavior, with broader distribution	Disappearance of *β-* and *γ-*relaxations upon mixing with MS	Stabilized during 1 month storage at ambient temperature	^16^
8–10 nm pores				
Celecoxib/MS	Unchanged α-relaxation behavior, with wider distribution. Rigid fraction faster (21 nm) and slower (2.5 nm)	-	Stabilized w/21 nm pores and destabilized w/2.5 pores	^26^
2.5 and 21 nm pores				
Celecoxib/MS	Unchanged α-relaxation behavior with broader distribution	-	Stabilized in the presence of 9–45% MS	^66^
23 nm pores				
Fenofibrate/MS	Accelerated mobility in center, suppressed on pore wall	γ-process disappeared	-	^67^
3 nm pores				
Flufenamic acid/MS	Highly mobile phase appeared in large pores (>7.1 nm, F-NMR)	-	Smaller pores exhibited stronger stabilization for entrapped molecules	^23^
MCM: 3.2 nm pores				
SBA: 7.1 nm pores				
MCF: 29 nm pores				
Ibuprofen/MS	Higher mobility	Slightly slower *γ-*relaxation at 203 K, *β-* and *γ-*relaxations faster in subglass region (159 K)	-	^24^
3.6 nm pores				
Ibuprofen/MS	Mobility increased (T1)	Faster mobility in larger pores (T2)	-	^68^
3.5 and 11.6 nm pores				
Indomethacin, Griseofulvin/Silicon	-	Limited mobility, T2 unchanged (ssNMR)	Stabilized for 4 months	^49^
11.1–13.3 nm pores				
Indomethacin/SBA-15	T1 increased, mobility decreased with increasing IDM concentration (ssNMR)	T2 decreased, mobility increased with increasing IDM concentration (ssNMR)	-	^69^
10 nm pores				
Itraconazole/SBA-15	-	T2 slightly increased at higher water content	-	^50^
6.3 and 8.3 nm pores				
Menthol/MS	Faster α-relaxation behavior with appearance of slow mobility (S-process) fraction	-	Stabilized in MCM pore	^21^
MCM: 3.2 nm pores				
SBA: 5.9 nm pores				
Naproxen/MS	Decrease in mobility w/decreasing pore size. Accelerated mobility in center, suppressed on pore wall for 5.9 and 3.2 nm pores	-	Most stabilized in 3.2 nm pore (6 years); Partially crystallized in 5.9 nm pore	^67,70^
MCM: 2.4 and 3.2 nm				
SBA: 5.9 nm				
Simvastatin/MS	Unchanged α-relaxation behavior at 9% MS	Unchanged *β-* and *γ-*relaxation behavior at 9% MS	Smaller particle size (2.5–3.7 μm) exhibited a stronger stabilization	^27^
23 nm pores				
Simvastatin/MS	Weak α-relaxation response at 33% drug	Weak γ-relaxation (hindered OH group mobility), unchanged *β-*relaxation	Stable for 3 years at ambient condition	^71^
3.5 nm pores				

In this study, β-relaxation of amorphous AAP was detected only with 10% D6P17, 10% D6P2 and 10% D4P2 (Figure 8a and Figure S10). These MSs were characterized by small pore sizes of 2.5 and 17 nm. The absence of this observation for MSs with 23 nm pores may be due to the too small amount of rigid fraction for MSs with 21 nm pores or their weaker confinement effect. The β-relaxation observed may reflect molecular motion of confined AAP, but other possibilities including interfacial heterogeneity can be assumed as its origin. Despite uncertainty of the origin of the secondary relaxation of AAP, the literature suggests that it stems from molecular motions that do not involve the entire molecule but were from a group larger than the size of −OH.^43^ In this study, compared with pure AAP, γ-relaxation was observed at slightly shorter relaxation times (lower τ_γ_) in 10% MS mixtures, indicating enhanced local mobility (Figure 9). Hydrogen bonds are expected to form between AAP molecules and silanol groups on the MS surface.^51,52^ These interfacial interactions compete with and disrupt the native hydrogen bonding network of the AAP, particularly the O–H···O=C and N–H···O interactions, both of which are important for constructing the crystal lattice.^53^ When AAP molecules are connected by these hydrogen bonds, each molecule has four neighboring molecules,^54^ with the OH group acting simultaneously as both a hydrogen bond donor and acceptor.^55^ Disruption of this network may alter local dynamics and contribute to faster γ-relaxation. A similar discussion has been provided for ibuprofen confined in MCM-41, where at lower temperatures, the γ-relaxation is accelerated due to increased free volume and the heterogeneous environment created by the pore structure.^24^ Under this assumption, molecules near the pore walls experience hydrogen bonding and restricted mobility, which can slow local motions, while those in the pore core retain greater freedom, contributing to faster γ-relaxation.

Secondary relaxations can arise from intramolecular (non-Johari–Goldstein: non-JG) or intermolecular (Johari–Goldstein: JG) motions. Non-JG relaxation involves the partial movement of the entire molecule and is characterized by lower energy barriers. In contrast, JG relaxation, which is considered a feature of all glass formers, is associated with small-angle reorientation of the entire molecule, exhibits a higher activation energy, and is regarded as a precursor to structural relaxation.^56^ For reference, JG-relaxations have been reported for β-adenosine, β-uridine, celecoxib, and telmisartan, with *E*_a_ values of 80, 77, 80, and 82 kJ/mol, respectively.^57–59^ Meanwhile, non-JG relaxations showed lower *E*_a_ values. For example, celecoxib, ezetimibe, nonivamide, fenofibrate, and ketoprofen were characterized by *E*_*a*_ values of 21, 38, 20, 33, and 38 kJ/mol, respectively.^60–63^ Therefore, the lower *E*_a_ observed in Figure 9 supports the assumption that secondary relaxation in AAP–MS systems is of the non-JG type and is not a precursor for structural relaxation. Another possible explanation is that it may be observation of collective motion, which is called slow Arrhenius process,^64^ as this process is also reasonable to explain the observed Arrhenius behavior and activation energy. In fact, this process seems to have relevance to the crystallization behavior.^65^

### 4.2 Effect of Particle and Pore Size of MS on the Stabilization of Overloaded Amorphous AAP

In this study, AAP was loaded beyond the total pore volume of the MS, forming an overloaded system in which a significant portion of the drug molecules resided outside the pores. Although investigations on the stabilization effect of MS have focused on confinement and surface interactions within the pores, recent studies suggest that MS can also stabilize external molecules, especially when appropriate interactions and efficient molecular exchange between the internal and external molecules are present.^26,27^ In this study, most AAP molecules remained as free fractions in the presence of 10% MSs, retaining their high molecular mobility outside the pores. In the presence of 10% D4P2 and 10% D6P2, both β- and γ-relaxation were detected. The presence of both relaxation modes was observed together with accelerated crystallization behavior (Figure 11), which may reflect influence of local mobility on the physical stability. For 50% MS mixtures, the behavior diverged based on pore size (Figure 11b). The 50% D3P21, 50% D6P21, 50% D9P21, and 50% D6P17 revealed a greater amount of intermediate fraction with slower α-relaxation, without any β- and γ-relaxation observed, indicating reduced molecular mobility. An increase in the melting enthalpy after storage (Figure 12) revealed that it was the intermediate fraction that underwent crystallization. This result indicates the importance of molecular exchange inside and outside the pores for stabilization.

In this study, a CRR-based explanation was provided to understand the pore size dependent stabilization behavior of the MS systems. In our previous studies, the superior stabilization effect of MS with 23 nm pores compared with that with 2.5 nm pores on celecoxib glass was explained by effective molecular exchange in the presence of large-pore MSs.^26^ This was considered one of the reasons for the different stabilization effects of MSs with different particle sizes (2.5–3.7 and 59 μm) on simvastatin glass, as they offer different surface areas.^27^ The MSs with large pores used in this study had a size of 21 nm, whereas the CRR size of pure AAP was 3 nm. Therefore, the pore size is much larger than the CRR size, and molecular exchange between the intermediate fraction inside and outside the pores may occur. Nucleation in the pores may become slower or hindered, presumably mainly because of the decrease in the CRR size, which may lower the nucleation temperature. Conversely, the pore sizes of D4P2 and D6P2 were comparable with that of the CRR. Considering presence of the rigid fraction on the surface of the pore walls, the pore size may become practically smaller than the CRR size. Therefore, molecular exchange should be significantly hindered, resulting in a limited stabilization effect on the molecules outside the pores. In this study, the impact of particle size differences was not found. The particle sizes used in this study (3, 6, and 9 μm) might not provide sufficient variation in the available surface area for molecular exchange to affect the crystallization.

The addition of MS to amorphous drugs has generally been found to either accelerate or retard crystallization. For example, the crystallization of celecoxib glass was accelerated in the presence of small-pore MS, whereas crystallization slowed down with large-pore MS.^26^ Upon the loading of a small amount of MS to amorphous aripiprazole, recrystallization was promoted; meanwhile, crystallization was inhibited at higher MS loadings (27–65%).^48^ The stabilization effect of MS has been explained by various mechanisms, including the confinement effect and surface interactions. However, no convincing explanation has yet been provided for this destabilization effect. In the present study, β- and γ-relaxation were associated with accelerated crystallization behavior in small pore MS systems, suggesting a possible role of local molecular mobility in the destabilization process.

Several studies have suggested a relationship between secondary relaxation and the crystallization of amorphous materials.^72–77^ For example, terahertz spectroscopy has demonstrated that JG secondary relaxation facilitates crystallization below the *T*_g_ for amorphous naproxen.^72^ A similar relationship between β-relaxation and physical stability has been observed using dynamic mechanical analysis, where a clear correlation between the *T*_g,β_ and onset of recrystallization was identified. Drugs with a lower *T*_g,β_, such as nifedipine, tend to crystallize faster than those with a higher *T*_g,β_.^73^ Furthermore, the characteristic crystallization time and β-JG relaxation time exhibited a correlation for indomethacin and celecoxib glasses,^74^ suggesting their relevance.

The crystallization to different crystal forms depending on the pore size is one of the curious observations in this study. In the presence of 2.5 nm pores, crystallization proceeded directly into form II, whereas form III was found in systems with larger pores (Figure 1A). Previous studies have shown that confinement can influence the polymorph selectivity. For example, a crystallization study of anthranilic acid in glass membranes with different pore sizes showed that the smaller pores favored nucleation of the metastable form II polymorph, while larger pores produced only form III. This behavior was attributed to the smaller critical nucleus size required for formation of form II under confined conditions.^78^ Similarly, nanoconfinement has been reported to stabilize different polymorphs of AAP depending on the confinement conditions. Crystallization of AAP in controlled porous glass (CPG) revealed stabilization of metastable form III in pores ranging from 43 to 103 nm.^79^ In addition, different pore morphologies were reported to influence both polymorph selection and solid–solid transitions of AAP. Straight cylindrical pores of anodic aluminum oxide favored the formation of form II and/or form III, whereas continuous isotropic pores in CPG formed form I under similar conditions.^80^ Pore sizes and solvent properties were also reported to influence polymorph outcomes in confined vortioxetine hydrobromide-silica nanopore systems.^81^ Therefore, in the present study, the 2.5 nm pore size may restrict the molecular packing and crystal growth required for Form III formation, thereby favoring direct crystallization into Form II. In addition, interaction between AAP molecules and silanol groups on the MS surface may disturb the hydrogen-bonding arrangement during nucleation and crystal growth, which could further influence polymorph selection. Such confinement and interfacial effects have also been reported to affect heterogeneous nucleation and polymorph behavior in the AAP system.^82^ However, the exact mechanism governing polymorph selection in AAP-MS systems remains unclear and requires further investigation.

### 4.3 Overall View on the Stabilization Effect of MS on Pharmaceutical Glasses

The crystal morphology and growth process were observed under a PLM to visualize the stabilization mechanism of AAP by MS. As shown in Figure 14, AAP crystals grew in spherical shapes under PLM in both the absence and the presence of MS with larger pores. However, in the presence of MS with smaller pores, the crystal shape became more irregular, most likely owing to the direct interaction of MS with the growth surface and reduced interfacial tension,^83^ which can also partially explain the faster crystal growth in the presence of MS. Multiple crystals formed frequently in the presence of MSs with smaller pores, suggesting enhanced nucleation. In our observation through PLM, crystals that appeared in the center of the field were captured for the analysis, which was easy in the presence of any type of MS. However, the observation for pure AAP was frequently disturbed by crystals grown from the edge of the cover glass, which occurred because of the long initiation time until the appearance of the spherical crystals. Thus, MSs with larger pores also likely enhanced nucleation as the appearance of crystals was faster than that of pure AAP. In summary, both the enhancement of nucleation and crystal growth in the presence of MSs were confirmed in the PLM studies.

Various confinement effects are summarized in Figure 15 to explain both the stabilization and destabilization effects. (1) if the pore size is smaller than the critical nucleus size, nucleation in the pores should be inhibited. This mechanism may be applicable only for MSs with small pores as the critical nucleus size is generally on the order of nanometers. However, assuming that the structure of molecules is influenced by the pore walls, this may also be observed for MSs with relatively large pores. (2) Steric hindrance in the pores also inhibits the growth of the crystals. The dimensions of the growth may also be influenced. (3) The CRR size decreases in the pores. Nucleation is expected to be enhanced when its critical size agrees with the CRR size.^83^ Therefore, a decrease in the CRR size may lower the nucleation temperature. (4) If the cross section is occupied by molecules, all of the molecules should be pulled by capillary condensation. This may accelerate both global and local motion and, therefore, may promote crystallization. (5) Rearrangement of the molecular interaction (mainly hydrogen bonding) network because of interaction with pore surfaces may produce regions with weak interaction, which may act as a slipping plane. It can accelerate both the global and local molecular mobility. Among these five mechanisms, the first two stabilize glasses. A decrease in the CRR size may result in both stabilization and destabilization. The last two destabilized the glass. The effect on stabilization differs depending on whether the drug is present only within the pores or outside. If the drug exists only inside pores, the smaller pores seem to be advantageous for stabilization; however, the small pores are not effective for stabilization outside pores. The enhancement effect of molecular mobility could potentially reduce stability. The stabilization of pharmaceutical glasses using mesoporous materials must be performed using a good strategy, as both stabilization and destabilization mechanisms exist depending on the combination of the drug and mesoporous materials, pore and particle sizes, and mixing ratio.

**Figure 15:**
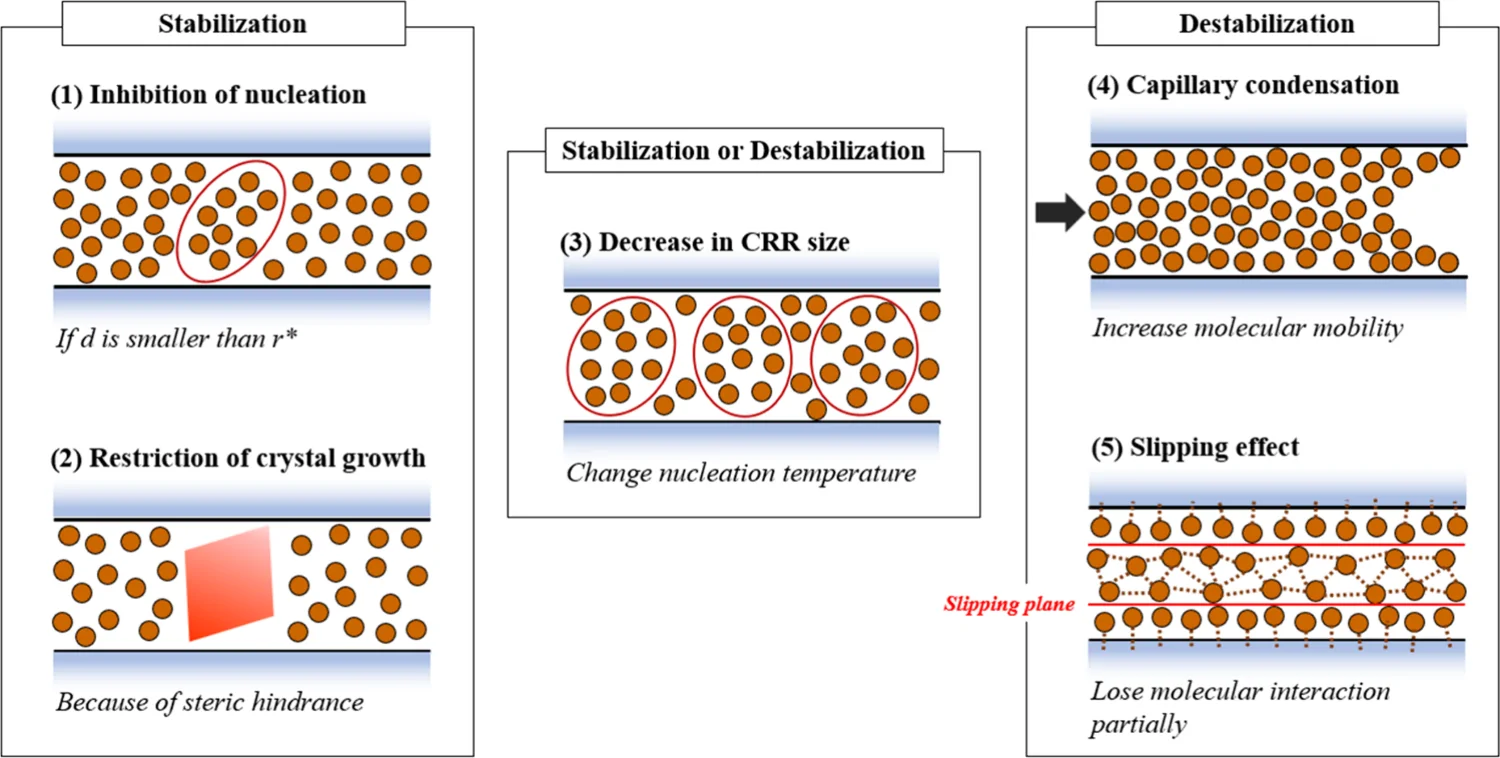
Confinement effect types of mesoporous materials.

## 5 Conclusion

The effects of MS particle and pore sizes on the physical stability of overloaded amorphous AAP were systemically investigated. Based on DSC analysis, the drug molecules were classified into free, intermediate, and rigid fractions. The intermediate fraction was likely involved for the enhanced stabilization observed in MSs with 17 and 21 nm pores. BDS measurements revealed that α-relaxation was slowed down in the presence of MSs, especially for MSs with large pores. Moreover, the appearance of β-relaxation with 2.5 and 17 nm pores and slight acceleration of γ-relaxation with all MSs were confirmed. In 10% MS mixtures, where almost all AAP molecules behaved as a free fraction, crystallization was accelerated in the presence of MSs with 2.5 nm pores. The accelerated crystallization observed in these systems occurred together with the appearance of β-relaxation and faster γ-relaxation, implying a possible involvement of local molecular mobility in the destabilization process. In 50% MS mixtures, MSs with 2.5 nm pores accelerated AAP crystallization in a similar manner. However, MSs with 17 and 21 nm pores significantly delayed crystallization, which was attributed to the crystallization of the intermediate fraction with slower molecular mobility. As the pore sizes of these MSs were larger than the CRR size of AAP, the observed stabilization effect was attributed to the possible ease of molecular exchange between inside and outside of the pores. Variations in particle size had no clear impact on the physical stability or molecular mobility of amorphous AAP. In summary, the addition of MSs can either accelerate or decelerate the molecular mobility; therefore, the crystallization of the guest molecules may also be accelerated or decelerated. If the pore size is larger than the CRR, the stabilization effect may become dominant because molecular exchange inside and outside the pores may occur more easily. However, if the pore size is comparable or smaller than this value, destabilization may dominate the system. These findings provide useful insights into the possible stabilization mechanism and formulation strategy for overloaded amorphous drugs using mesoporous silica.

## Supplementary Material



## Notes

TGA and DSC first heating curves of pure AAP and its mixture with 25% MS, dielectric loss spectra of pure AAP and mixtures containing 25%, 50%, 60%, and 70% MS, correlation between Avrami constant and intermediate fraction, as well as CRR size values calculated from DSC data (PDF)
