# Supplementary material for: Systematic Investigation on Particle and Pore Size Effects of Mesoporous Silica on Molecular Mobility and Physical Stability of Overloaded Amorphous Acetaminophen
Source: Mol Pharm. 2026 Aug 24;23(9):4656–72. doi: 10.1021/acs.molpharmaceut.6c00402 (PMC13556133; doi:10.1021/acs.molpharmaceut.6c00402)
Supplement: Supplementary file 1 [file mp6c00402_si_001.pdf]

Supporting information for

Systematic Investigation on Particle and Pore Size Effects of Mesoporous Silica on Molecular Mobility and Physical Stability of Overloaded Amorphous Acetaminophen

Francisca Larasati[1,2], Kohsaku Kawakami[1,2,*]

[1] Research Center for Macromolecules and Biomaterials, National Institute for Materials Science, 1-1 Namiki, Tsukuba, Ibaraki 305-0044, Japan

[2] Graduate School of Science and Technology, University of Tsukuba, 1-1-1 Tennodai, Tsukuba, Ibaraki 305-8577, Japan

* Corresponding author

E-mail: kawakami.kohsaku@nims.go.jp, Tel. +81-29-860-4424

1. **DSC first heating curve for pure AAP and its mixture with 25% MS**

   Figure S1 below presents the first heating curve of the samples. Two melting peaks were visible for the mixtures with 21 nm pores. The lower temperature of melting peak with reduced intensity suggests the influence of confinement effect of the pore[1].

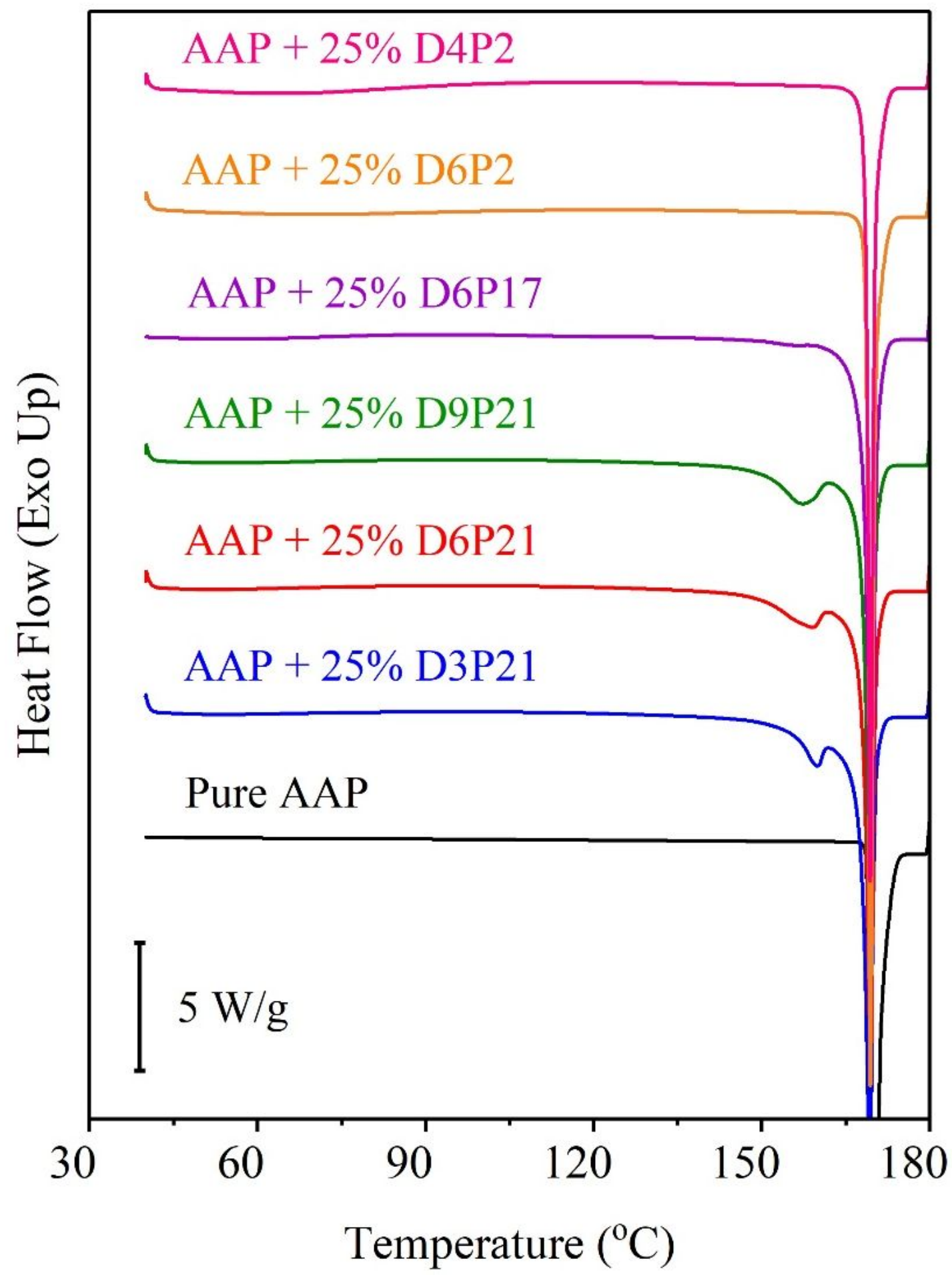

Figure S1. The first heating DSC curves for pure AAP and its mixture with 25% MS

2. Thermogravimetric analysis of pure AAP and its mixtures with 25% MSs.

Figure S2 shows thermogravimetric analysis results for pure AAP and its mixture with MSs. Pure AAP showed almost complete decomposition before reaching 300 °C. AAP-MSs mixtures exhibited residual masses consistent with the MS content. By focusing on the weight decrease starting from the endset temperature of the weight loss at ca. 290 °C, the fraction of the highly stabilized AAP molecules was calculated (Table S1).

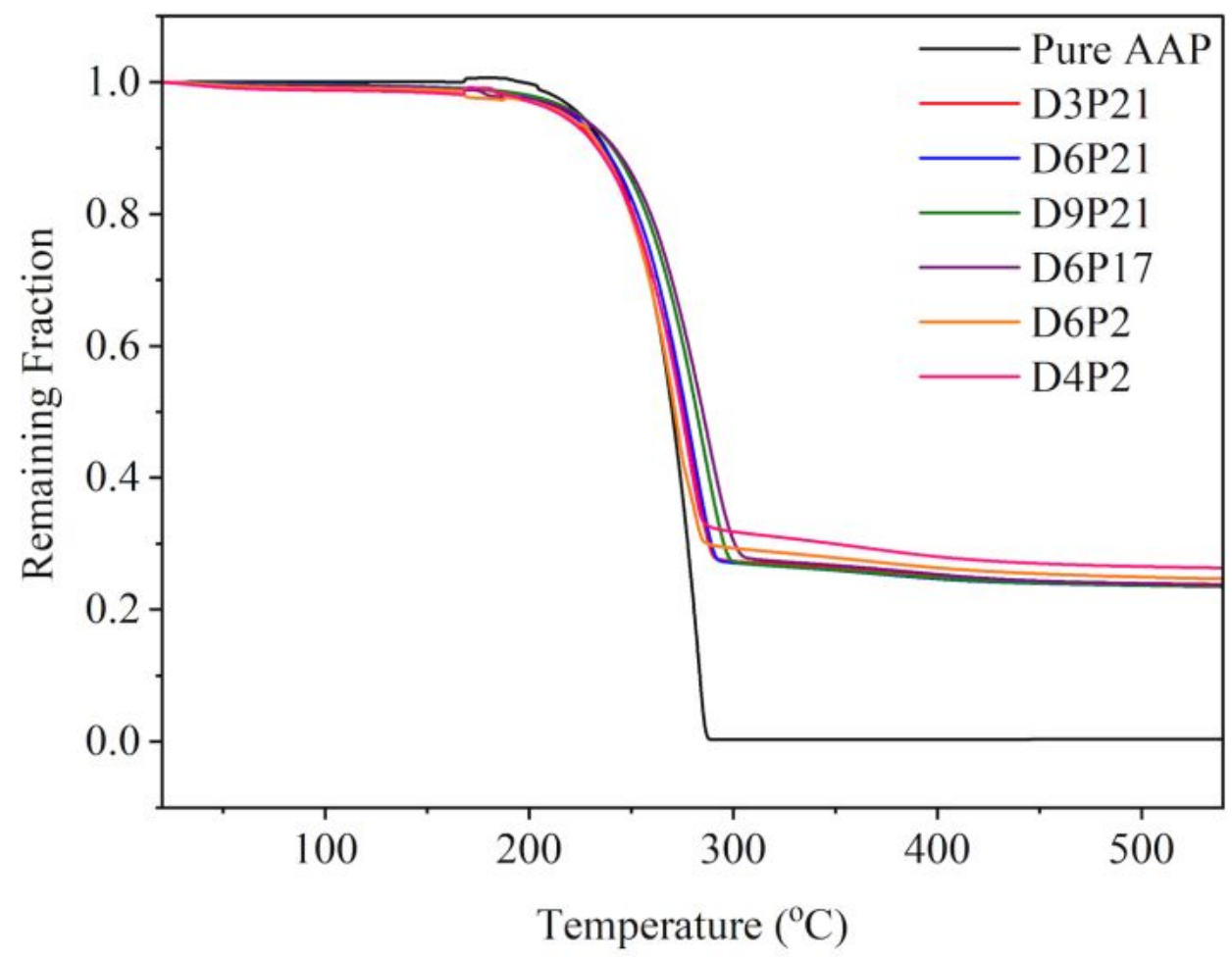

Figure S2. Thermogravimetric curves of pure AAP and its mixtures with 25% MSs.

Table S1. Estimated amount of stabilized AAP molecules from TGA and DSC

Sample Name	Stabilized Molecules from TGA (%)	Stabilized Molecules from DSC (Rigid Fraction) (%)
D3P21	3.98	3.74
D6P21	4.12	6.50
D9P21	3.94	5.78
D6P17	4.28	7.58
D6P2	5.96	17.62
D4P2	6.83	20.90

3. Dielectric loss spectra of pure AAP and 25, 50, 60, 70% MS mixtures

The dielectric loss spectra for mixtures of AAP with 10%, 25%, 50%, 60%, 70%, and % MS required to achieve complete confinement of AAP molecules are presented below. γ-relaxations were observed in 10% MS, while distinct β-relaxations were observed only in the sample with 2.5 nm and 17 nm pores at ratio 10% and 25% MS. For other MS ratios, only α-

relaxation was detected. As the MS content increased, the dielectric response decreased, which is attributed to the reduced AAP fraction in the system[2].

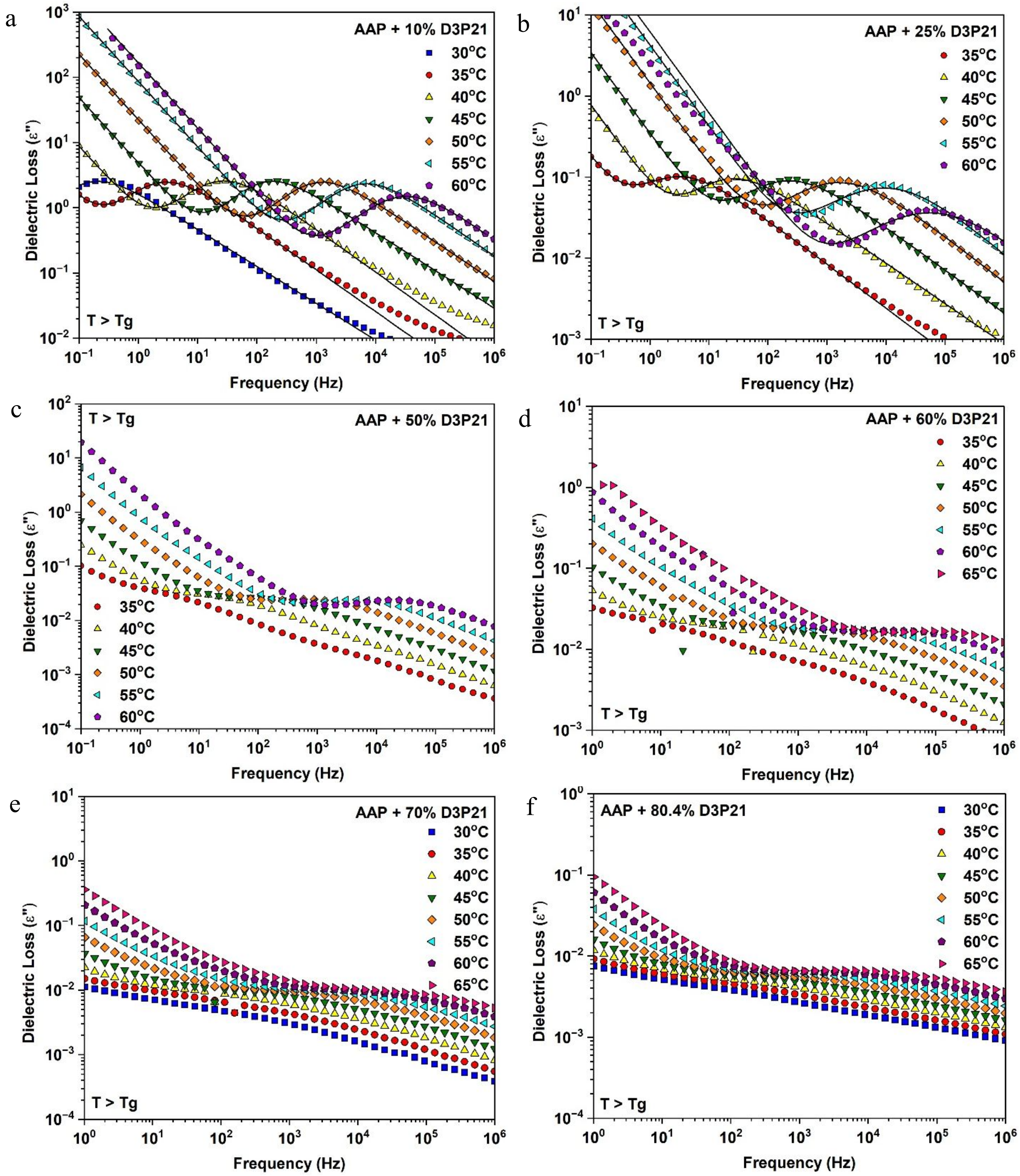

Figure S3. Dielectric loss spectra of mixtures of pure AAP and D3P21 at different ratios (a-f), measured at temperatures above Tg to investigate structural relaxation.

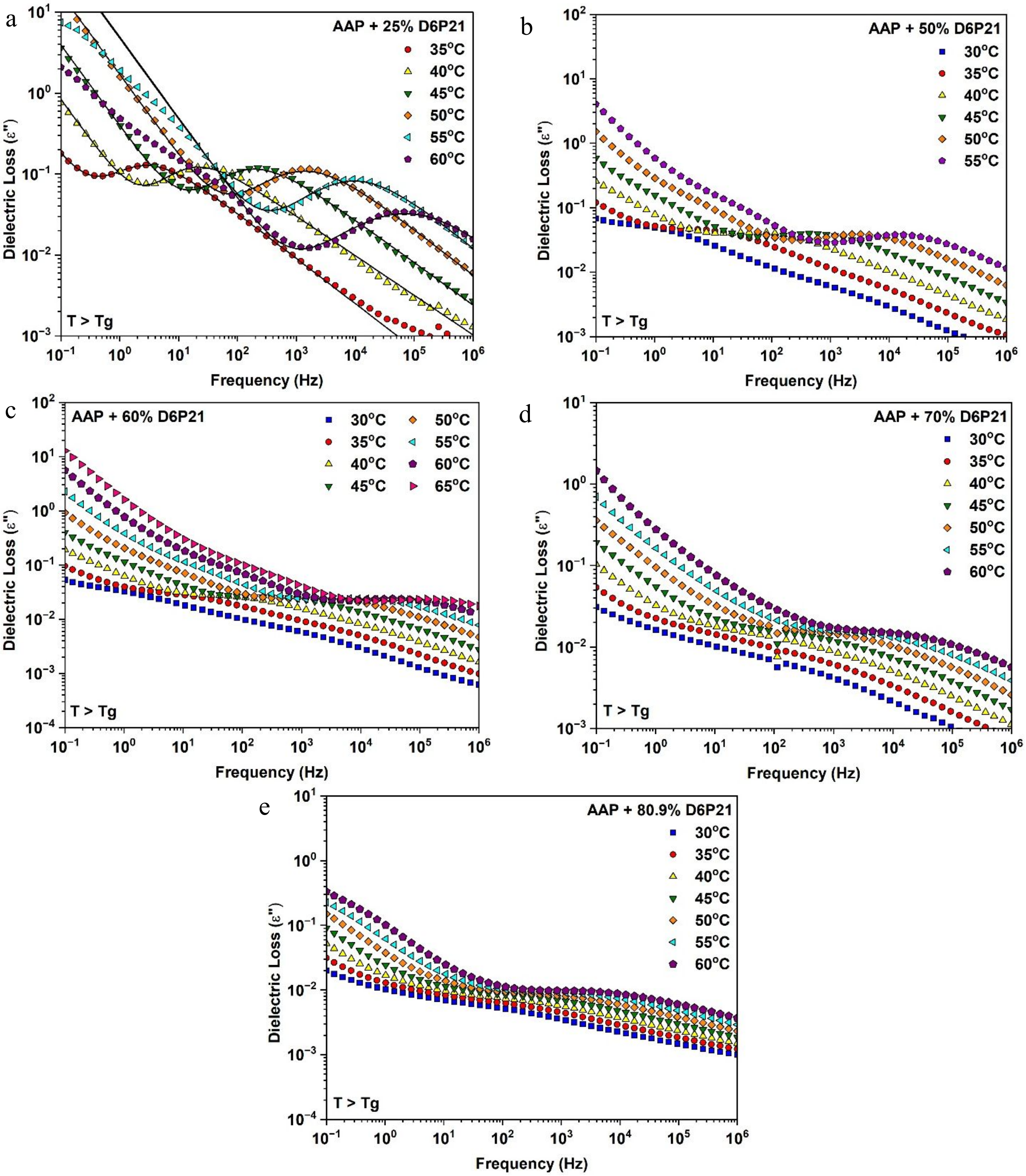

Figure S4. Dielectric loss spectra of mixtures of pure AAP and D6P21 at different ratios (a-e), measured at temperatures above Tg to investigate structural relaxation.

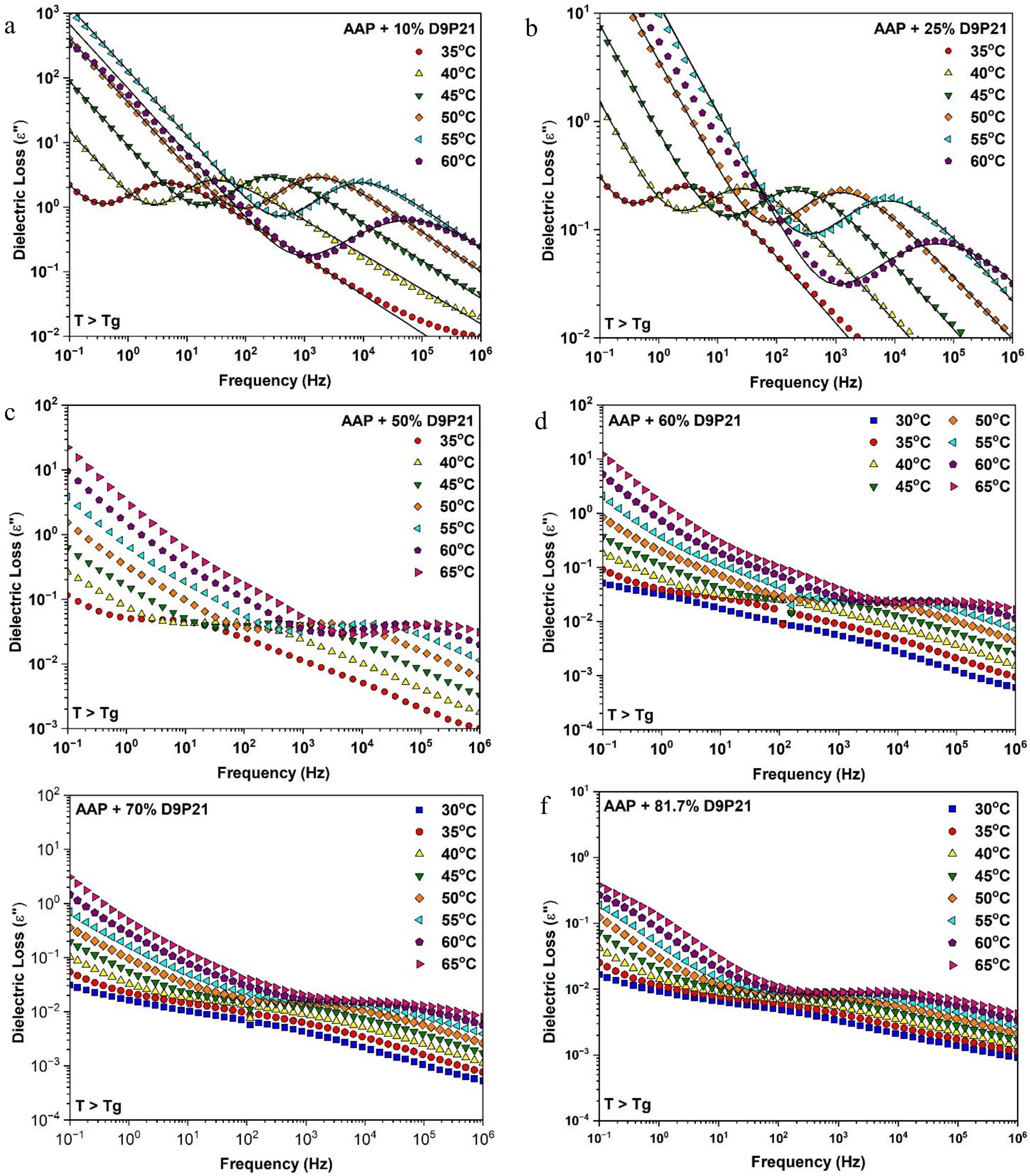

Figure S5. Dielectric loss spectra of mixtures of pure AAP and D9P21 at different ratios (a-f), measured at temperatures above Tg to investigate structural relaxation.

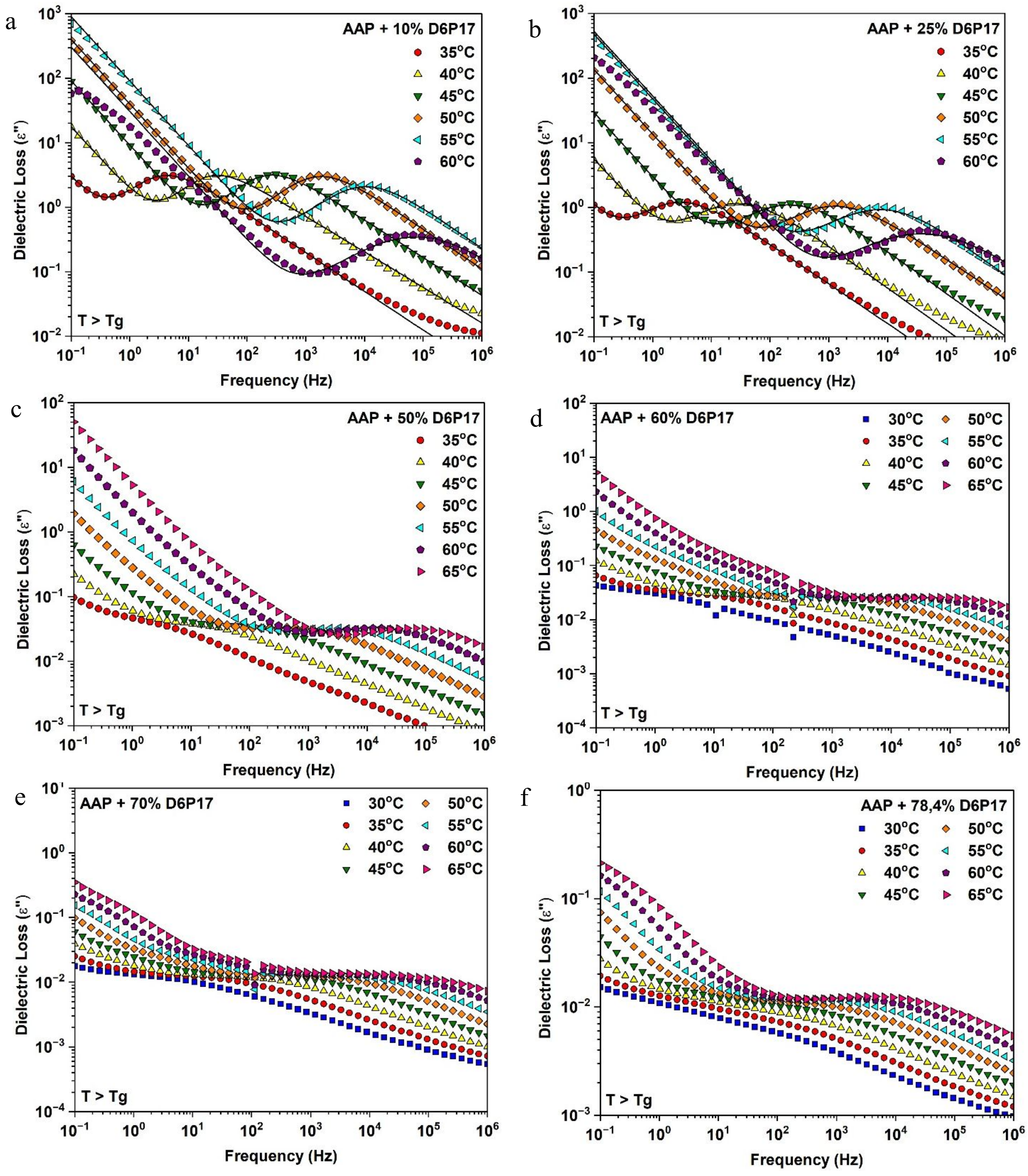

Figure S6. Dielectric loss spectra of mixtures of pure AAP and D6P17 at different ratios (a-f), measured at temperatures above Tg to investigate structural relaxation.

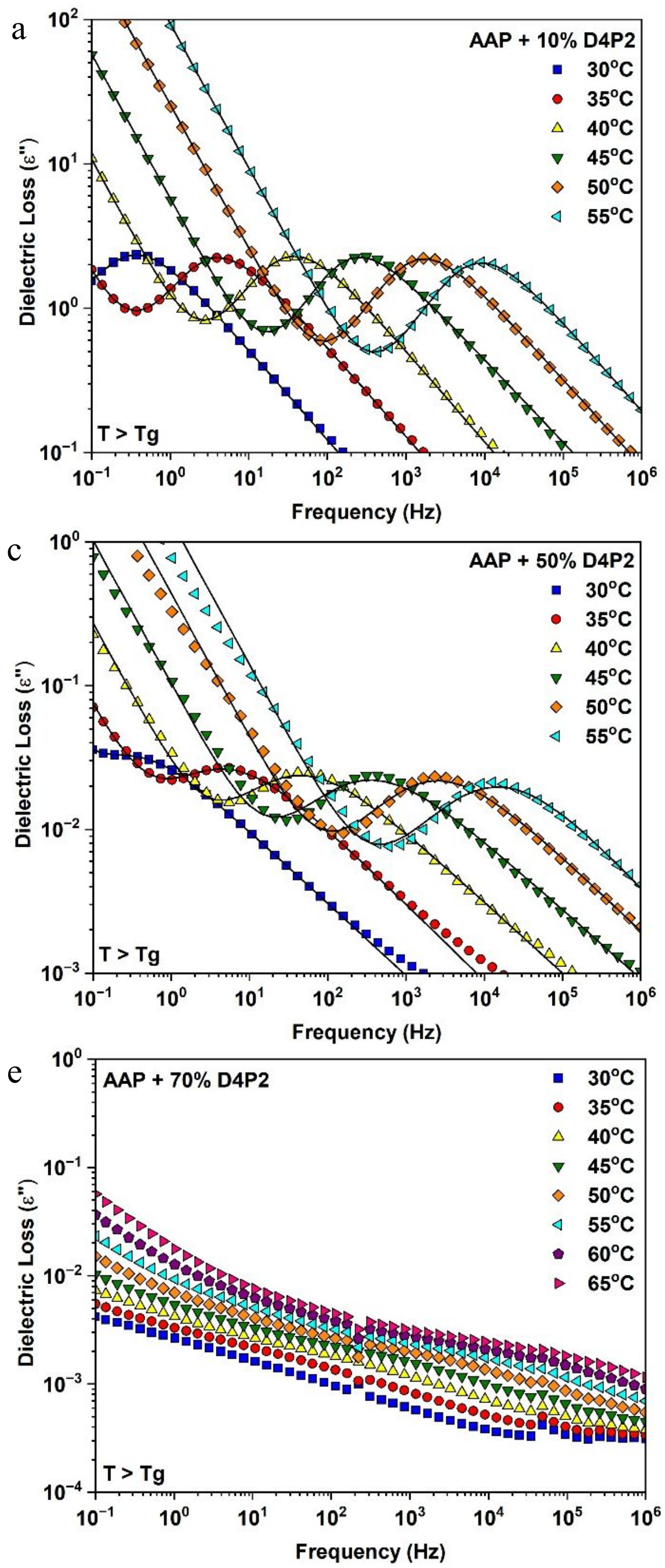

Figure S7. Dielectric loss spectra of mixtures of pure AAP and D4P2 at different ratios (a-f), measured at temperatures above Tg to investigate structural relaxation.

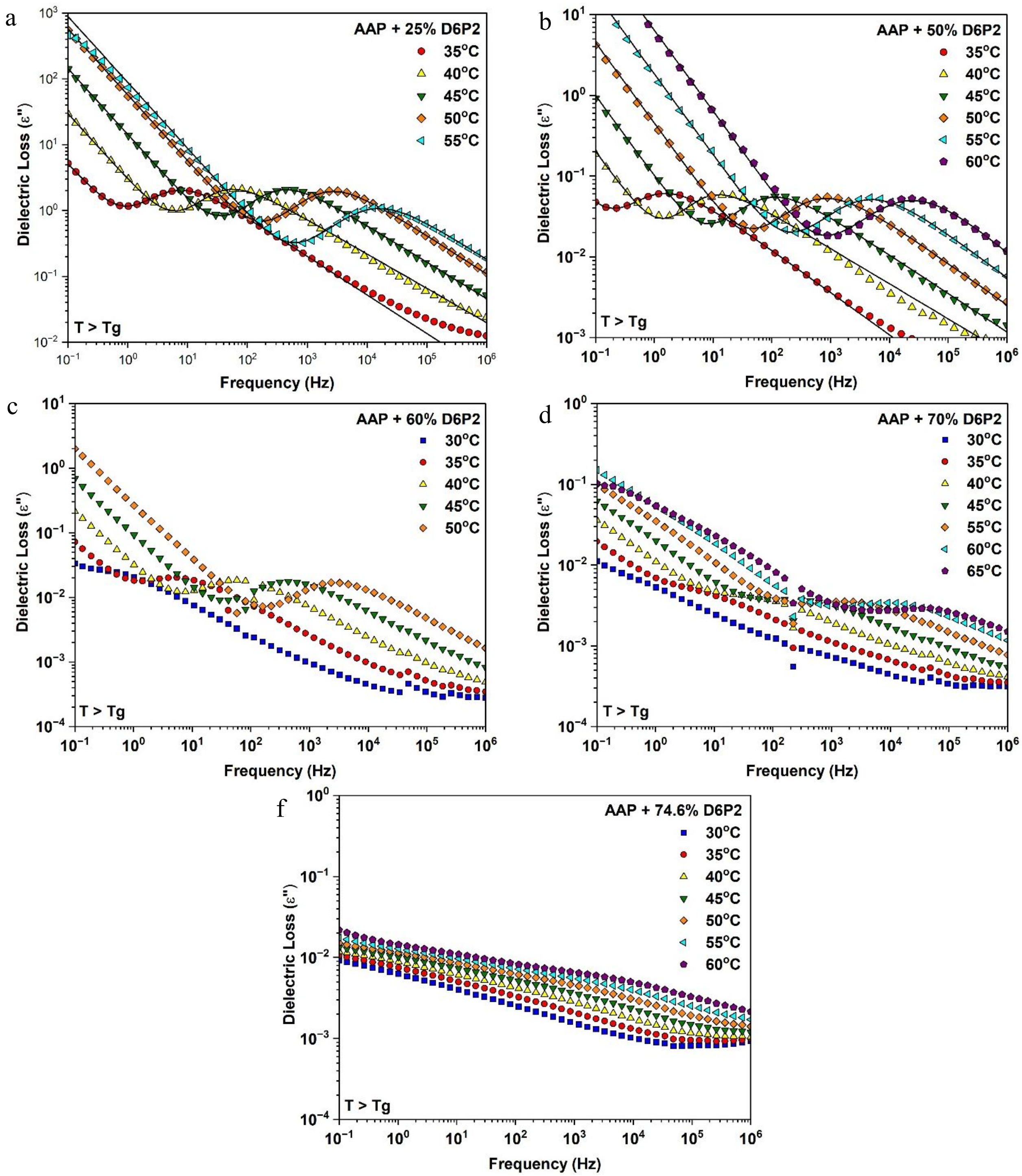

Figure S8. Dielectric loss spectra of mixtures of pure AAP and D6P2 at different ratios (a-e), measured at temperatures above Tg to investigate structural relaxation.

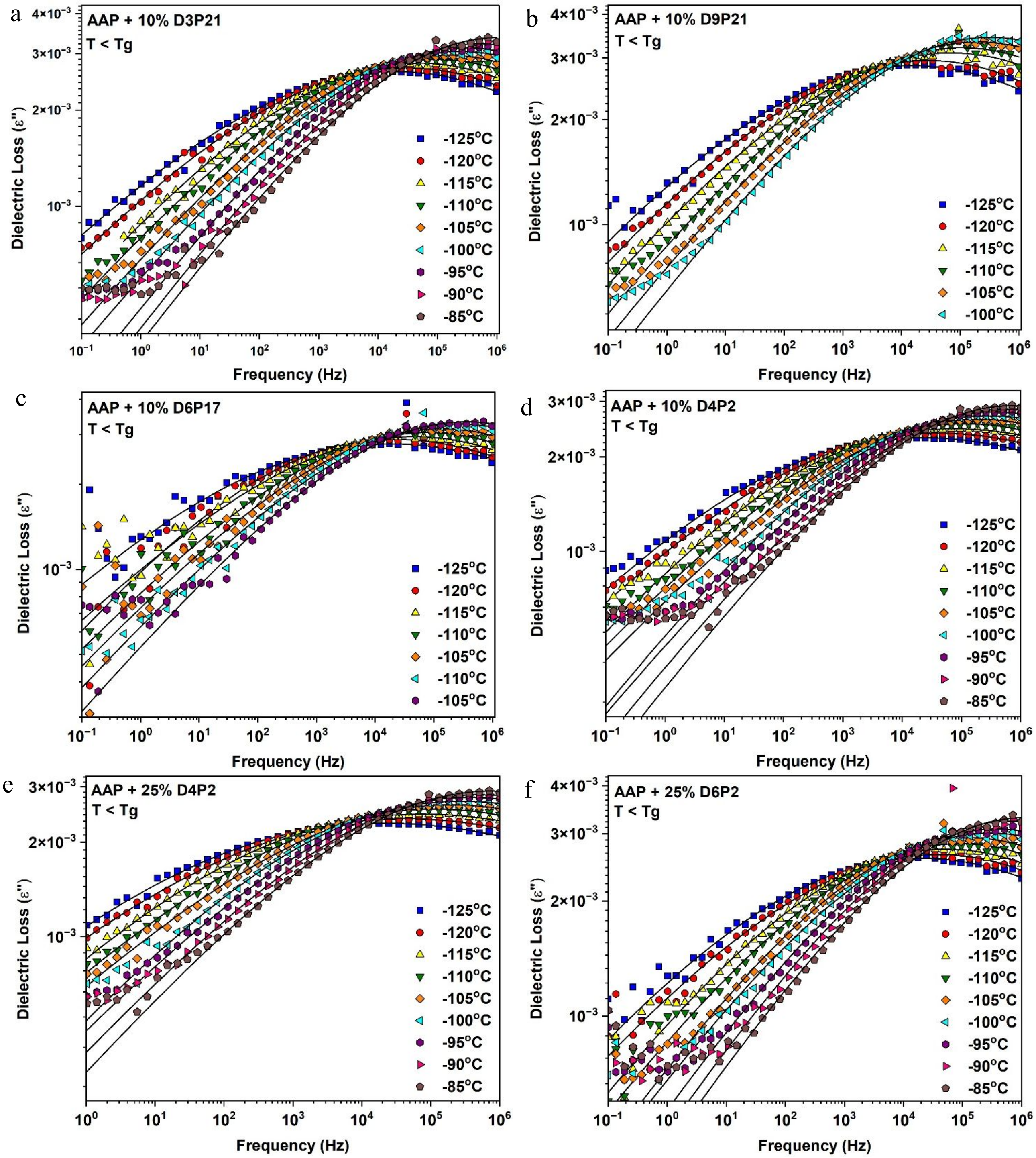

Figure S9. Dielectric loss spectra of mixtures of pure AAP with D3P21, D9P21, D6P17, D4P2 and D6P2 at different ratios (a-f), measured at temperatures below Tg to investigate secondary relaxation (γ-relaxation).

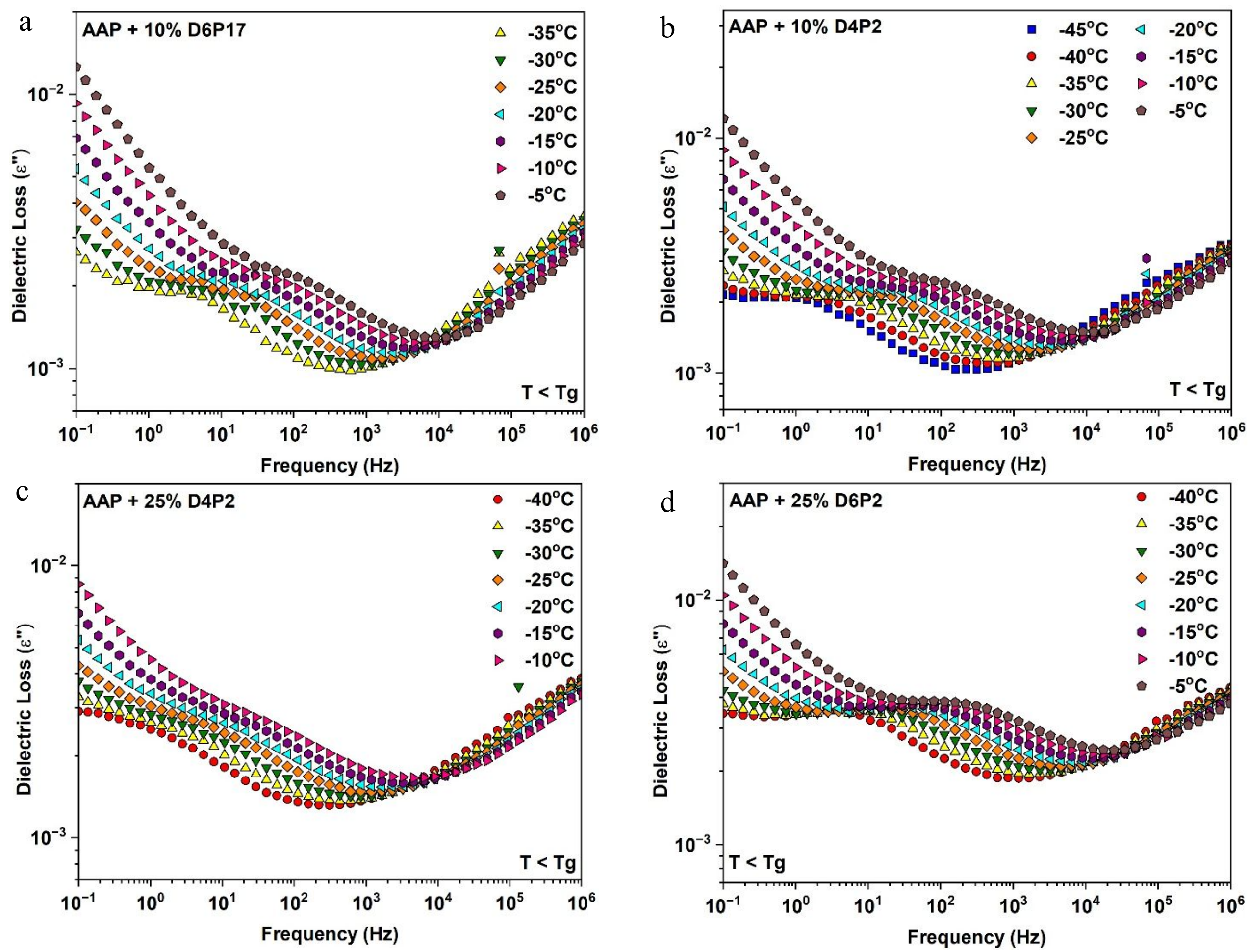

Figure S10. Dielectric loss spectra of mixtures of pure AAP with D6P17, D4P2, and D6P2 at different ratios (a-d), measured at temperatures below Tg to investigate secondary relaxation (β-relaxation).

4. Correlation between Avrami constant and intermediate fraction

Mixtures with larger intermediate fractions tended to exhibit reduced n values, suggesting a decrease in dimension of crystal growth and/or an increase in the heterogeneity of crystallization.

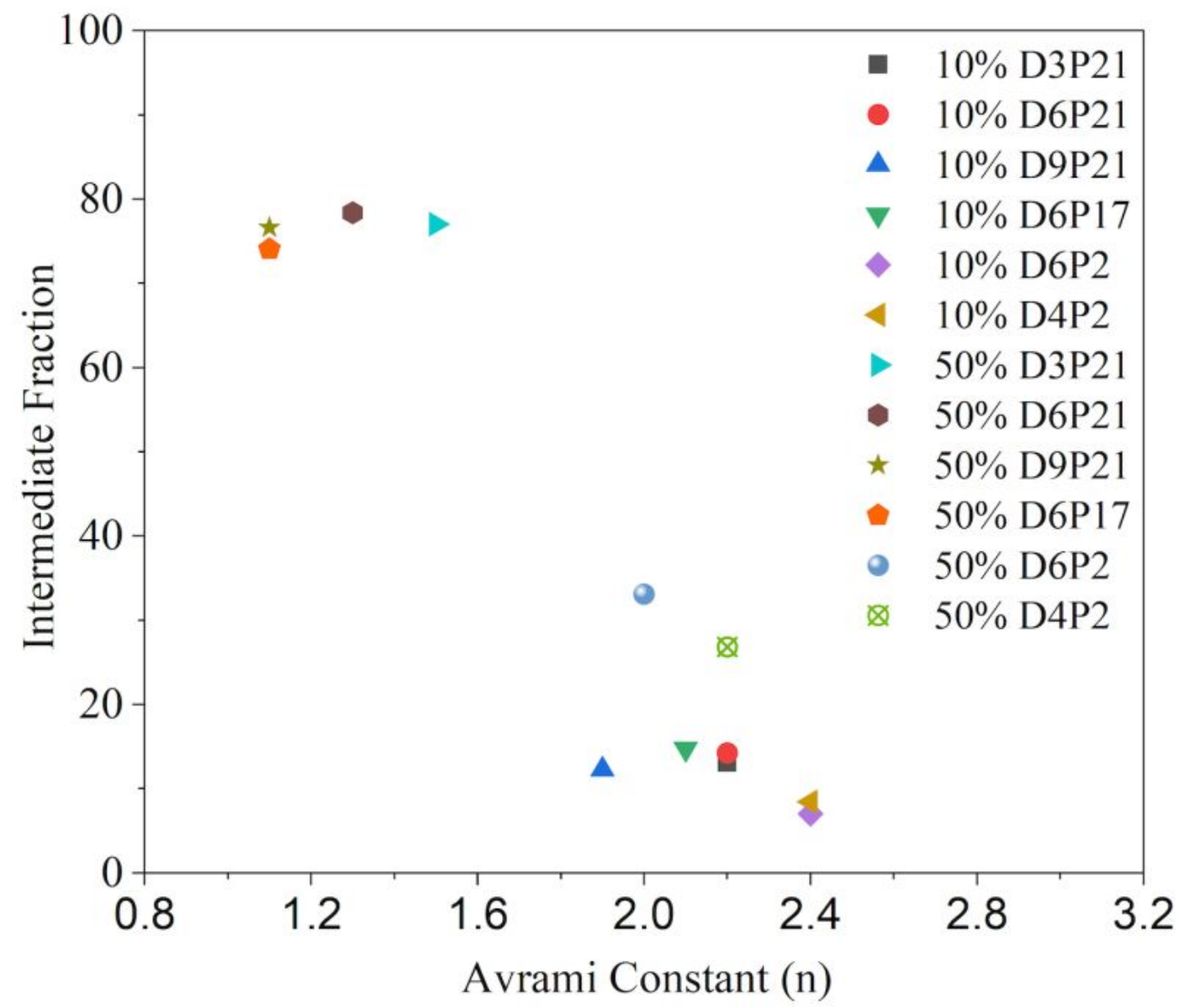

Figure S11 Correlation between Avrami constant and intermediate fraction

5. Size of CRR calculated from DSC

The size of the CRR was calculated by observing its glass transition behavior using DSC. Approximately 5 mg of pure AAP and its mixtures with MS were loaded into Tzero pans and sealed. The samples were heated to 180 °C at a heating rate of 10 °C/min for melting, followed by cooling to 0 °C at a programmed rate of 50 °C/min. Subsequently, the samples were heated at a rate of 10 °C/min to observe the glass transition. The measurements were repeated on three different samples. The CRR size (L) was calculated using the following equation[3]:

$$L = \left\{ \frac{3kT_g^2}{4\pi\rho\Delta d^2{}_{T_g}} \left(\frac{1}{C_{pg}} - \frac{1}{C_{pl}} \right) \right\}^{\frac{1}{3}}$$

where k is Boltzmann's constant, T_g is the onset glass transition temperature, and ρ is the density. Δd_{Tg} is the half of the glass transition width determined from the difference between the onset and endpoints of T_g. C_{pg} and C_{pl} are the specific heat capacities of the glass and supercooled liquid, respectively.

The influence of MS addition on the CRR size of amorphous AAP (calculated from DSC) is shown in Figure S12. The addition of MS decreased the CRR size regardless of the MS types. In larger pores, the addition of MSs decreased the CRR size, while the size remained constant with the further addition of MSs with 2.5 nm pores.

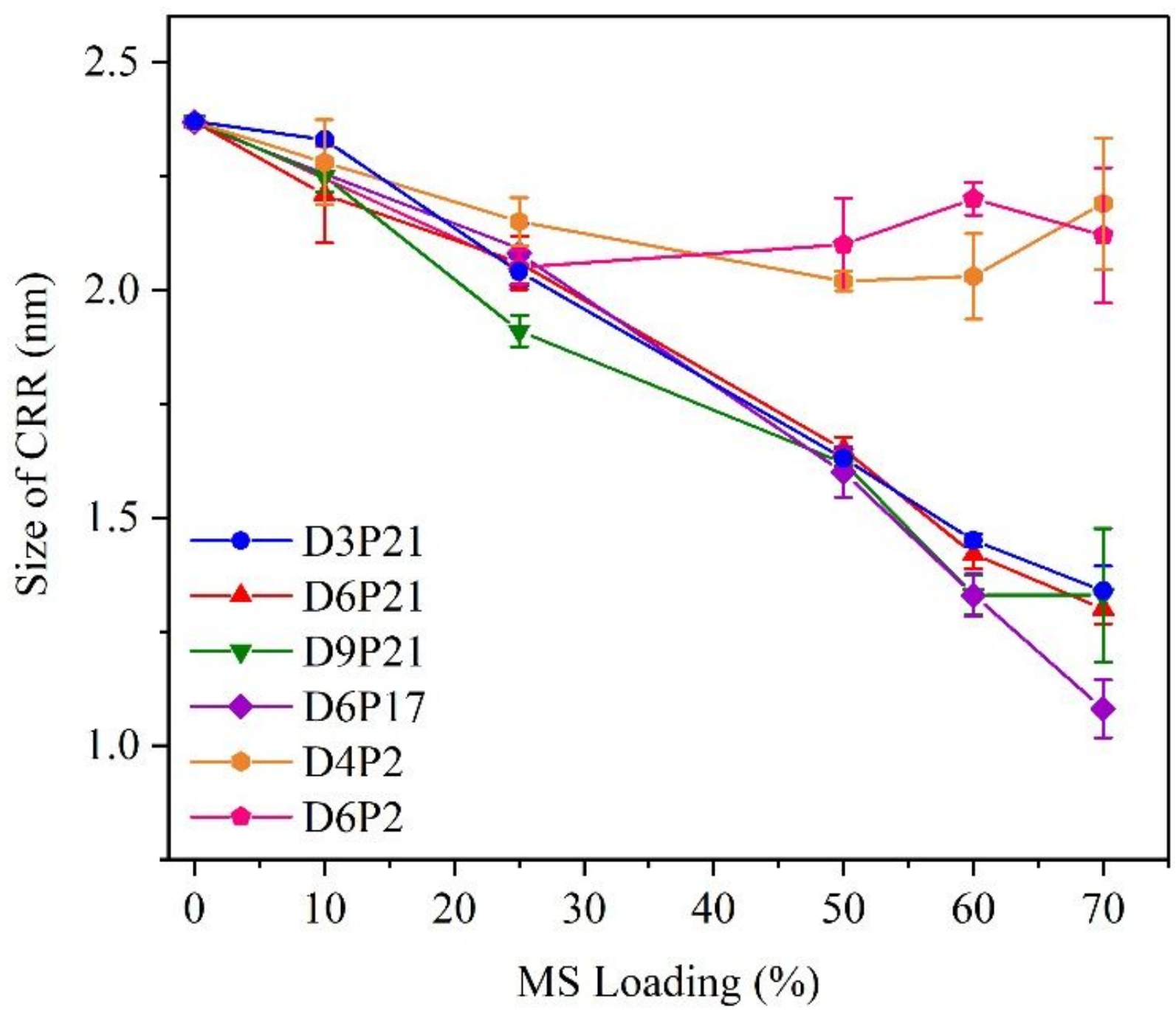

Figure S12. Sizes of the CRR of AAP in mixtures with MSs as a function of the MS loading, determined from DSC.

References

1. Kaptay, G. The Gibbs Equation versus the Kelvin and the Gibbs–Thomson Equations to Describe Nucleation and Equilibrium of Nanomaterials. *J. Nanosci. Nanotechnol*. **2012**, 12 (3), 2625–2633.
2. Kramarczyk, D.; Knapik-Kowalczuk, J.; Klimontko, J.; Kurek, M.; Jachowicz, R.; Paluch, M. Tuning the Physical State of Aripiprazole by Mesoporous Silica. *Mol. Pharmaceutics* **2024**, 21 (5), 2315–2326.
3. Donth, E. The Size of Cooperatively Rearranging Regions at the Glass Transition. *J. Non-Cryst. Solids* **1982**, 53, 325-330.
